# Phosphatidic acid-dependent localization and basal de-phosphorylation of RA-GEFs regulate lymphocyte trafficking

**DOI:** 10.1186/s12915-020-00809-0

**Published:** 2020-06-29

**Authors:** Sayaka Ishihara, Tsuyoshi Sato, Guangwei Du, Daniele Guardavaccaro, Akihiko Nakajima, Satoshi Sawai, Tohru Kataoka, Koko Katagiri

**Affiliations:** 1 Department of Biosciences, School of Science, Kitasato University, 1-15-1 Kitasato, Minamiku, Sagamihara, Kanagawa 252-0344 Japan; 2 Department of Integrative Biology & Pharmacology, University of Texas Health Science at Houston, 6431 Fannin St, Houston, TX 77030 USA; 3 Hubrecht Institute-KNAW and University Medical Center Utrecht, Uppsalalaan 8, 3584 CT Utrecht, The Netherlands; 4 Department of Basic Science, Graduate School of Arts and Sciences, University of Tokyo, Tokyo, 153-8902 Japan; 5 Division of Molecular Biology, Department of Biochemistry and Molecular Biology, Kobe University Graduate School of Medicine, 7-5-1 Kusunoki-cho, Chuo-ku, Kobe, 650-0017 Japan

**Keywords:** Rap1, Lymphocyte trafficking, Chemokine, Migration

## Abstract

**Background:**

Lymphocytes circulate between peripheral lymphoid tissues via blood and lymphatic systems, and chemokine-induced migration is important in trafficking lymphocytes to distant sites. The small GTPase Rap1 is important in mediating lymphocyte motility, and Rap1-GEFs are involved in chemokine-mediated Rap1 activation. Here, we describe the roles and mechanisms of Rap1-GEFs in lymphocyte trafficking.

**Results:**

In this study, we show that RA-GEF-1 and 2 (also known as Rapgef2 and 6) are key guanine nucleotide exchange factors (GEF) for Rap1 in lymphocyte trafficking. Mice harboring T cell-specific knockouts of *Rapgef2/6* demonstrate defective homing and egress of T cells. Sphingosine-1-phosphate (S1P) as well as chemokines activates Rap1 in a RA-GEF-1/2-dependent manner, and their deficiency in T cells impairs Mst1 phosphorylation, cell polarization, and chemotaxis toward S1P gradient. On the other hand, B cell-specific knockouts of *Rapgef2/6* impair chemokine-dependent retention of B cells in the bone marrow and passively facilitate egress. Phospholipase D2-dependent production of phosphatidic acid by these chemotactic factors determines spatial distribution of Rap1-GTP subsequent to membrane localization of RA-GEFs and induces the development of front membrane. On the other hand, basal de-phosphorylation of RA-GEFs is necessary for chemotactic factor-dependent increase in GEF activity for Rap1.

**Conclusions:**

We demonstrate here that subcellular distribution and activation of RA-GEFs are key factors for a directional movement of lymphocytes and that phosphatidic acid is critical for membrane translocation of RA-GEFs with chemokine stimulation.

## Background

Lymphocytes recirculate continually between the peripheral lymphoid tissues via the blood and lymphatic systems [1]. Lymphocytes enter across the high endothelial venule (HEV) into lymphoid tissues, and egress from efferent lymphatic vessels, return to the blood. When rolling lymphocytes are exposed to chemokines present on HEV, chemokine signaling coupled with Gα_i_ proteins activates leukocyte function associated-1 (LFA-1), a major receptor that mediates homing to peripheral lymph nodes. In a previous study, we showed that Rap1, which is rapidly activated by chemokines, is indispensable for LFA-1-dependent transmigration across the HEV [2, 3]. The RAPL (regulator of adhesion and cell polarization enriched in lymphoid tissues)-Mst1/2 (Mst) (mammalian Ste-20 like protein kinase) complex, downstream effectors of Rap1, induces cell polarization with LFA-1 clustering on the front membrane, which is critical for Rap1-GTP-mediated migration [4, 5]. Furthermore, we recently demonstrate that Rap1-GDP retains cell rigidity through activation of lymphocyte-orientated kinase and phosphorylation of ezrin-moesin-radixin [6]; thereby, the conversion to Rap1-GTP also releases these restraints.

Sphingosine-1-phosphate receptor 1 (S1PR1) is also a Gα_i_ protein-coupled receptor (GPCR) and expressed in mature single-positive CD4 or CD8 thymocytes, and conditional deletion of S1PR1 in T cells alone was sufficient to block their egress from the thymus [7]. As S1P provides a critical chemotactic cue, and levels of S1P are high in the blood and lymph and low in most tissues, it was postulated that this “S1P gradient” would play a role in lymphocyte egress [8]. S1PR1 is transduced via multiple downstream pathways and results in diverse biological outcomes [9]. Mice deficient for the Rap1 downstream effectors RAPL or Mst1/2 demonstrated impaired thymic egress [10, 11], suggesting that S1P activates Rap1.

Conversion between GTP- and GDP-bound states is controlled by guanine nucleotide exchange factors (GEFs) and GTPase-activating proteins (GAPs) [12]. In particular, GEFs enhance the formation of the GTP-bound active conformation in response to upstream signals mediated by various cell surface receptors such as chemokine receptor. Although previous studies suggested that several signaling pathways such as Abl (Abelson)-CRK (chicken tumor virus number 10 regulator of kinase) or JAKs (Janus kinases)-RhoA pathways are partially involved in chemokine-dependent Rap1 activation [13–16], which GEFs are critical for homing and egress of primary T and B cells remains unsolved.

To date, various GEFs for Rap1 have been identified in mammalian cells [12]. C3G (Crk SH3 domain-binding guanine nucleotide-releasing factor) binds to the adaptor protein Crk, which is involved in Abl tyrosine kinase-dependent activation of Rap1. Epac (exchange factor directly activated by cAMP), cAMP-GEF is activated through direct association with cAMP. CalDAG-GEFI (calcium and diacylglycerol (DAG)-regulated guanine nucleotide), which contains calcium- and diacylglycerol-binding motifs, has a role in Rap1 activation in response to these second messengers. RA-GEF-1 (also termed PDZ-GEF1 or Rapgef2) and RA-GEF-2 (also termed PDZ-GEF2 or Rapgef6) contain putative CNB (cyclic nucleotide-binding), REM (Ras exchange motif), PDZ (Postsynaptic density 95, PSD-95; Discs large, Dlg; Zonula occludens-1, ZO-1), and RA (Ras/Rap association) domains as well as the GEF catalytic domain [17–19]. RA-GEFs were reported to associate with phosphatidic acid (PA) [20].

Recently, we reported PA is critical for membrane translocation of RA-GEFs with chemokine stimulation [21]. PA plays important roles in cell proliferation, survival, and migration. PLD2 has been proven to be mainly located and generate PA at the plasma membrane by external stimuli using a newly developed PA biosensor, phosphatidic biosensor with superior sensitivity (PASS) [22, 23].

Here, we exhibit that RA-GEFs are major GEFs in both chemokine and S1P-mediated Rap1 activation. GPCR-dependent signaling induces PLD2-dependent PA synthesis and recruitment of RA-GEFs to the plasma membrane, which determine the generation sites of Rap1-GTP and produce the front adhesive membrane of the migrating cell. We also suggest that the suppression of RA-GEFs by phosphorylation is constitutively canceled by de-phosphorylation. This basal de-phosphorylation is indispensable for chemokine-dependent activation of Rap1. Thus, we are the first to identify Rap1-GEFs critical for lymphocyte trafficking in vivo and to shed light on the regulatory mechanisms of RA-GEFs as a directional indicator in cell locomotion.

## Result

### RA-GEF deficiency impairs chemokine-dependent Rap1 activation and T cell homing in peripheral lymph nodes

**Fig. 1:**
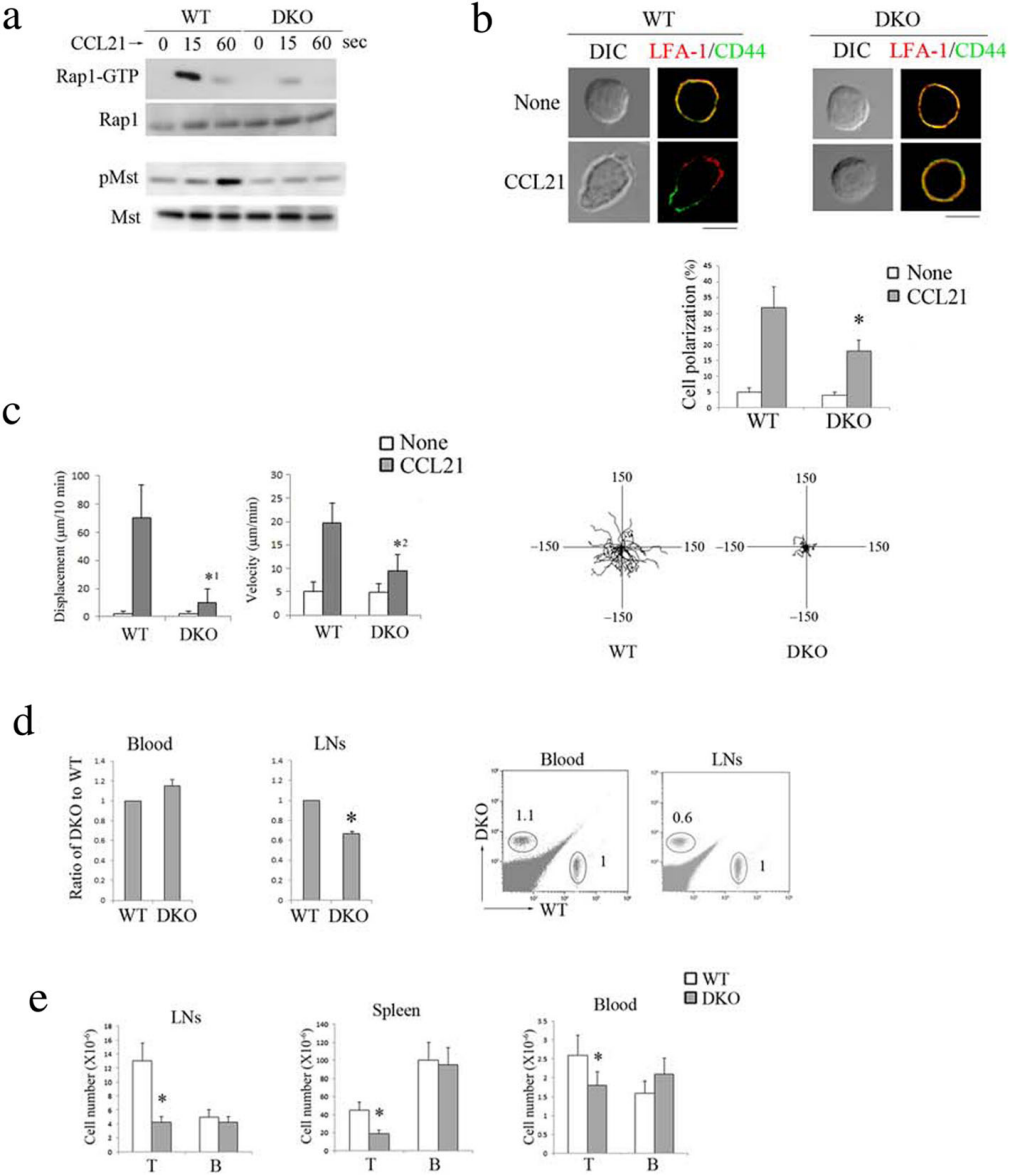
Deficiency of RA-GEF in T cells impaired Rap1 activation and homing. **a** (Top) GTP-bound Rap1 was analyzed by a pull-down assay using GST-Ral-GDS-RBD. Wild-type (WT) or RA-GEF-deficient (DKO) T cells were stimulated with CCL21 at the indicated times. Bound Rap1 (Rap1-GTP) and total Rap1 were detected with anti-Rap1. (Bottom) Phosphorylation (p-) of Mst1/2 was examined with anti-phosphorylated Mst1/2. Total Mst1 is shown below. **b** (Top) WT and DKO T cells were stimulated with CCL21 for 10 min at 37 °C. The cells were stained with anti-LFA-1, alexaFluor 633 anti-Rat Ig, and FITC-CD44. DIC, differential interference contrast. Scale bar, 5 μm. Cells with segregated LFA-1 and CD44 accompanied with elongated cell shapes were considered to be polarized cells. (Bottom) The graph shows the percentages of the polarized cells (*n* = 50). *P < 0.001 versus WT cells. **c** (Left) Displacement and velocity of WT and DKO T cells were measured on ICAM-1 with or without CCL21 (*n* = 50). *^1^*P* < 0.001, *^2^*P* < 0.002 versus WT T cells. (Right) Representative tracks of WT or DKO T cells on the ICAM-1 in the presence of CCL21 are shown. Each line represents a single-cell track. **d** WT and DKO T cells were labeled with CFSE and CMTMR, respectively, and injected into normal mice. After 1 h, lymphocytes from injected mice were analyzed. (Left) Ratios of the number of DKO cells in the blood or lymph nodes relative to WT cells (adjusted to 1) (*n* = 3). *P < 0.001 versus corresponding cells. (Right) Representative flow cytometry profiles are shown. The numbers indicate the ratio of DKO to WT cells. **e** The numbers of T cells and B cells from the lymph nodes, spleen, and blood from WT or DKO mice are shown (*n* = 10). Cells were stained with anti-CD3, anti-B220, and anti-CD45 and analyzed using the flow cytometry. *P < 0.001 versus WT mice. Each bar graph represents the means ± SEM

Since T cells expressed both RA-GEF-1 and RA-GEF-2 (Additional file 1: Figure S1A), and single knockout did not affect Rap1 activation induced by chemokine (Additional file 1: Figure S1B), we deleted both in T cells (RA-GEF-deficient T cells). RA-GEF-1 conditional knockout (*Rapgef2*^f/f^: *Lck-Cre* or *CD4-Cre*), *Rapgef*6^+/−^ mice were crossed with *Rapgef2*^f/f^, *Rapgef6*^*+/−*^ mice to obtain the *Lck-cre* or *CD4-cre/Rapgef2*^*f/f*^*/Rapgef6*-null mice (RA-GEF DKO). These mice grew normally without gross abnormalities. Littermate mice (*Rapgef2*^f/f^, *Rapgef6*^*+/+*^) (wild-type; WT) were used as control mice. There was no difference in all data described in this paper between *Lck-cre* and *CD4-cre/Rapgef2*^*f/f*^*/Rapgef6*-null mice. In a first set of experiments, we assessed the influence of RA-GEF-1/2 (RA-GEF) deficiency on chemokine-dependent Rap1 activation. Rap1-GTP levels in RA-GEF-deficient T cells were reduced to 14% of T cells derived from control littermate mice at the peak time point after CCL21 stimulation (Fig. 1a). Mst phosphorylation, which was induced in a Rap1-GTP-dependent manner after stimulation with CCL21, also decreased to less than 14% of control cells (Fig. 1a).

In a previous study, we demonstrated that Rap1 activation is essential for cell polarization with segregated membranes in which LFA-1 accumulated at the leading edge and CD44 at the uropod [4]. As shown in Fig. 1b, polarized cells accompanying the appearance of the front membrane with LFA-1 clustering were significantly reduced in RA-GEF-deficient T cells after CCL21 stimulation. Therefore, we examined the effect of RA-GEF deficiency on the CCL21-dependent migration of T cells on the intracellular adhesion molecule 1 (ICAM-1)-coated plate. The displacement is the shortest distance the cells migrated from the initial to the final position and reflects a directional persistence. The displacement of RA-GEF-deficient T cells was reduced by approximately 16% when compared to WT T cells (Fig. 1c). Velocity is the displacement per unit time in a given direction and reflects speed with a direction. The average velocity was also reduced by approximately 48% in RA-GEF-deficient T cells (Fig. 1c). Thus, in response to CCL21, RA-GEF deficiency impaired T cell polarization and LFA-1-dependent migration on the ICAM-1.

We then examined the effects of RA-GEF-1/2 deficiency on T cell homing. WT and RA-GEF-deficient naïve T cells (CD62L^hi^CD44^lo^CD69^−^) were labeled with different fluorescent dyes and adaptively transferred into WT mice. The RA-GEF-deficient T cells showed a defective homing capacity into peripheral lymph nodes (Fig. 1d). Consistent with that, the T cell number in the peripheral lymph nodes of RA-GEF DKO mice significantly diminished (Fig. 1e). T cell numbers in the spleen and blood of RA-GEF DKO mice were also reduced, relative to WT mice (Fig. 1e), suggesting an impairment in the supply of mature T cells from the thymus.

### RA-GEF deficiency leads to the accumulation of mature SP cells in the thymus

**Fig. 2:**
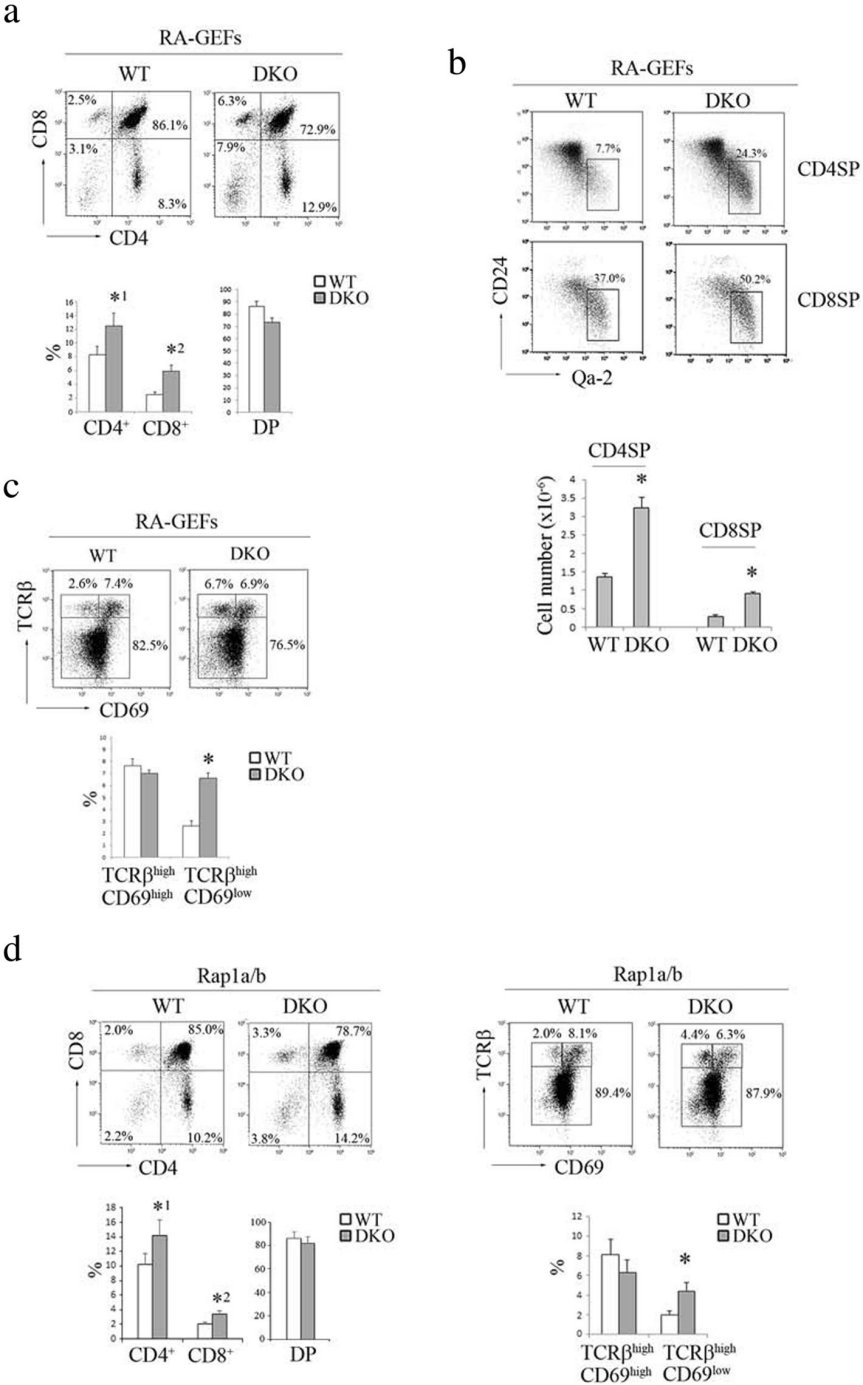
Defective thymic egress in RA-GEF DKO mice. **a** (Top) Representative CD4 and CD8 profiles of the thymus from WT or DKO mice. (Bottom) Percentages of CD4^+^CD8^+^ double-positive (DP), CD4^+^ or CD8^+^ single-positive cells from the thymi of WT or DKO mice (*n* = 10). *^1^*P* < 0.002, *^2^*P* < 0.001 versus corresponding WT cells. **b** (Top) Representative CD24 and Qa-2 profiles on CD4^+^ or CD8^+^ single-positive cells from the thymus of WT or DKO mice. (Bottom) The numbers of CD24^low^ and Qa-2^high^, CD4^+^ or CD8^+^ single-positive (SP) cells from the thymi of WT or DKO mice (*n* = 5). *P < 0.001 versus corresponding WT cells. **c** (Top) Representative CD69 and TCRβ profiles of the thymus from WT or DKO mice. (Bottom) Percentages of TCRβ ^high^ CD69^low^ and TCRβ ^high^ CD69^high^ cells in thymocytes of WT or DKO mice (*n* = 5). *P < 0.001 versus corresponding WT cells. **d** (Left upper) Representative CD4 and CD8 profiles of the thymus from WT or Rap1a and b double knockout (DKO) mice. (Left lower) Percentages of CD4^+^CD8^+^ DP, CD4^+^ or CD8^+^ SP cells from the thymi from WT or Rap1a/b DKO mice (*n* = 10). *^1^*P* < 0.005, *^2^*P* < 0.002 versus corresponding WT cells. (Right upper) Representative CD69 and TCRβ profiles of the thymus from WT or Rap1a/b DKO mice are shown. (Right lower) Percentages of TCRβ ^high^ CD69^low^ and TCRβ ^high^ CD69^high^ cells from the thymi of WT or Rap1a/b DKO mice (*n* = 10). *P < 0.001 versus corresponding WT cells. Each bar graph represents the means ± SEM

T cell development was assessed by measuring the surface expression of CD4 and CD8. CD4 or CD8 single-positive cells (SP) in RA-GEF DKO mice increased by approximately 1.6-fold (Fig. 2a). The newly generated SP underwent a multistage maturation in the thymic medulla. The emigration process followed this maturation [24]. The Qa-2 antigen was used to identify a subset of the mature SP. A subset of SP with a low expression of CD24 was more mature than one with a high expression of CD24 [24]. Specifically, the frequency of CD4 SP and CD8 SP with a mature phenotype (Qa-2^+^CD24^lo^) increased from 7.7 to 24.3% and from 37.0 to 50.2%, respectively. Their total cell number increased by twofold in RA-GEF DKO mice, compared to WT mice (Fig. 2b). This result suggests that the egress process of mature SP might be impaired in RA-GEFDKO mice.

S1PR1 signaling is essential for T cell egress from the thymus. CD69 physically interacts with S1PR1, resulting in the inhibition of the surface S1PR1 expression. Therefore, we examined whether RA-GEF deficiency affects the expression of CD69. TCRβ^high^CD69^low^ mature SP increased twofold in the RA-GEF DKO mice, suggesting that the accumulation of mature SP in the thymus of RA-GEF DKO mice was not due to defective regulation of S1PR1 (Fig. 2c).

T cell-specific Rap1a/b-double knockout mice (Rap1a/b DKO) also demonstrated an abnormal accumulation of SP in the thymus and an increased ratio of TCRβ^high^CD69^low^ mature SP (Fig. 2d). These results suggest that RA-GEF-dependent Rap1 activation in mature SP is required for emigration from the thymus.

### RA-GEF are involved in S1P-dependent Rap1 activation and locomotion along the S1P gradient

**Fig. 3:**
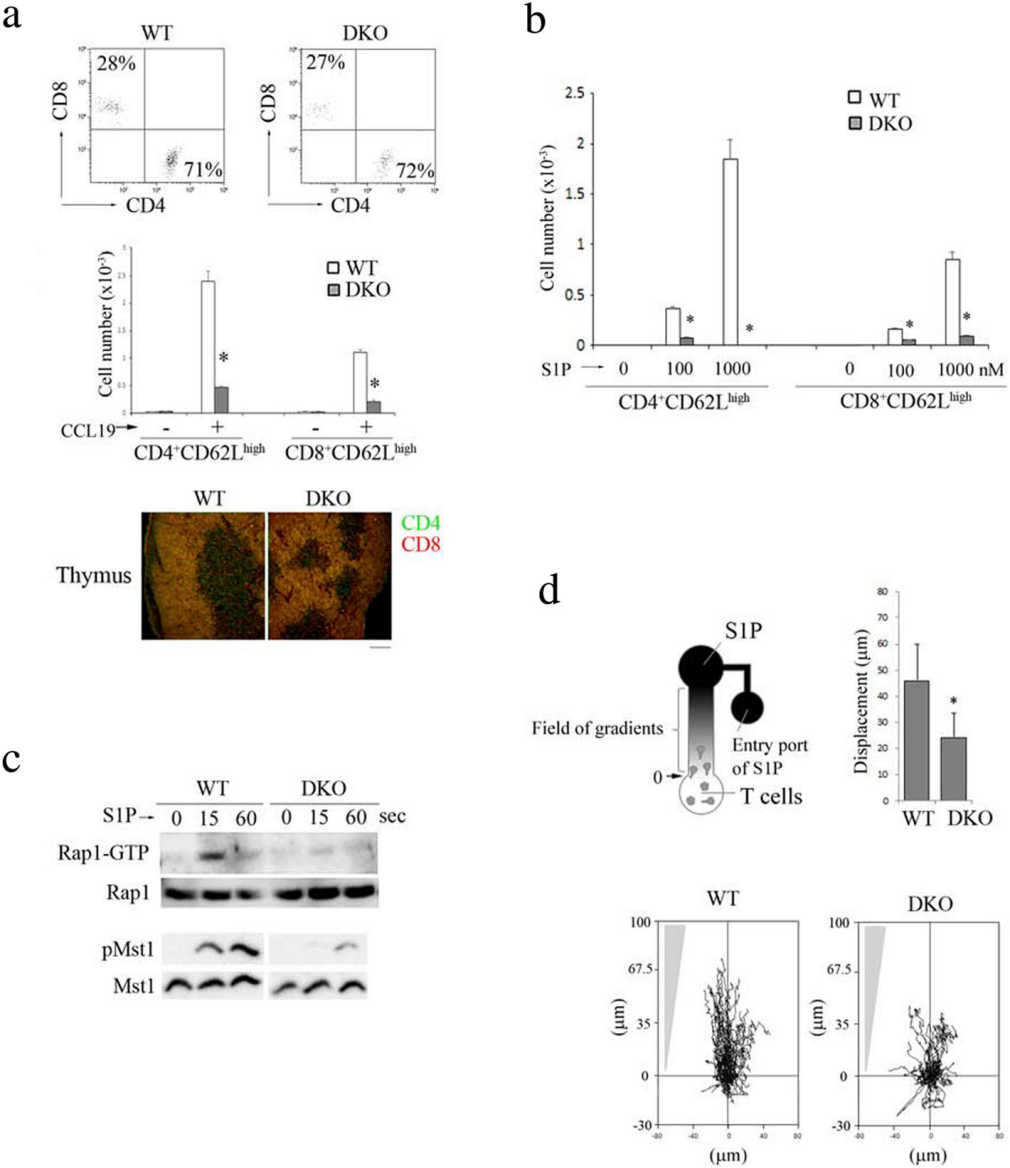
S1P activates Rap1 in RA-GEF-1/2-dependent manner, which is critical for chemotaxis toward S1P gradient. **a** (Top) Emigration of thymocytes toward CCL19 from the thymus lobes in Transwell chemotactic chambers. CD4 and CD8 profiles of CD3-gating cells that emigrated from WT and RA-GEF DKO thymus after 3 h of incubation with CCL19 are shown. (Middle) The numbers of emigrated cells from three independent experiments using WT or DKO mice. *P < 0.001 versus corresponding WT cells. (Bottom) Thymus sections from WT and DKO mice stained for CD4 (green) and CD8 (red). Scale bar, 200 μm. **b** The migration of thymocytes toward S1P in Transwell chemotactic chambers. The cell numbers of CD4^+^ or CD8^+^, and CD62L^high^ cells that migrated toward S1P after 3 h of incubation with S1P (*n =* 3). *P < 0.001 versus corresponding WT cells. **c** (Top) WT or DKO T cells were stimulated with 1 μM of S1P for the indicated times, lysed, and subjected to a pull-down assay. Bound Rap1 (Rap1-GTP) and total Rap1 were detected with anti-Rap1. (Bottom) Phosphorylation (p-) of Mst1/2 in WT or DKO T cells stimulated with 1 μM of S1P for the indicated times was examined with anti-phosphorylated Mst1/2. Total Mst1 is shown below. **d** (Top left) The experimental set-up of scheme. Time-lapse sequences of WT and DKO T cells migrating toward the S1P source were recorded. (Top right) Displacement of WT and DKO T cells (*n* = 30). *P < 0.001 versus WT T cells. (Bottom) Representative tracks of WT or DKO T cell on ICAM-1 in response to S1P gradient are shown. Each line represents a single-cell track. Each bar graph represents the means ± SEM

To investigate the effects of RA-GEF-1/2 deficiency on the emigration of SP from the thymus, we examined chemokine-dependent thymocyte migration in Transwell assays using thymic lobes. In response to the CCL19, which is a CCR7 ligand, CD4 and CD8 SP from RA-GEF DKO mice displayed severe defects during migration from the thymus (Fig. 3a). Consistent with this result, immunohistology using confocal analysis of frozen thymus sections demonstrated a defect in the medullary accumulation of SP thymocytes (Fig. 3a). Thus, the RA-GEF-1/2 deficiency is likely to reduce the mature SP cells’ approach to the thymus egress sites.

Higher amounts of S1P in the blood can stimulate S1PR1 on mature thymocytes to induce egress from the thymus. Next, we examined the ability of SP thymocytes to migrate in response to the high level of S1P. WT-mature SP thymocytes (CD4^+^ or CD8^+^, CD62L^high^) progressively migrated toward S1P. However, migration toward S1P was eliminated in the RA-GEF-deficient mature SP thymocytes (Fig. 3b). These data suggest that S1P-stimulated migration is also dependent on the activation of RA-GEF.

This finding led us to examine whether S1P activates Rap1 through RA-GEF. As shown in Fig. 3c, Rap1-GTP in RA-GEF-deficient T cells was reduced to 18% of the WT cells after S1P stimulation. The phosphorylation of Mst1 was also reduced to less than 17% of WT cell levels (Fig. 3c).

Because the S1P gradient plays a vital role in egress, we analyzed the effects of RA-GEF-1/2 deficiency on T cell locomotion along the S1P gradient. To this end, we developed the novel device, which formed stable gradients in S1P using the chemotaxis chamber (Additional file 2: Figure S2). The value of the gradient profile in this chemotaxis chamber was determined through visual observation using a fluorescein dye (Additional file 2: Figure S2). As shown in Fig. 3d, control T cells demonstrated directional migration toward the S1P gradient, whereas RA-GEF-deficient T cells could not move to the constant direction. These results show that RA-GEF in T cells is required for sensing the S1P gradient.

### The RA-GEF’s deficiency in B cells reduces the retention of immature B cells in the bone marrow, resulting in the acceleration of B cell egress from the bone marrow

**Fig. 4:**
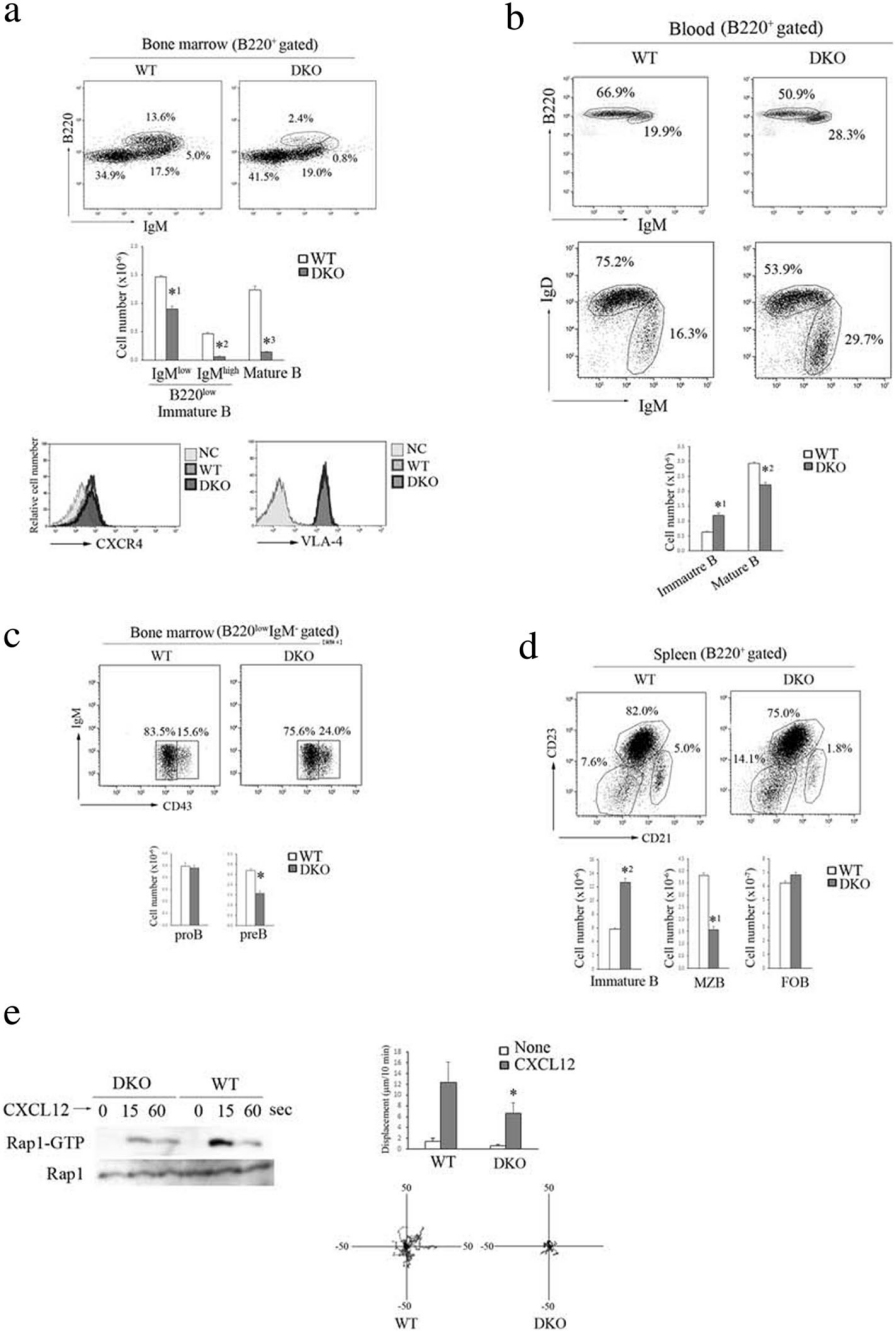
Impaired retention of RA-GEF-deficient B cells within the bone marrow. **a** (Top) Representative B220 and IgM profiles of B220^+^-gated bone marrow cells from WT or RA-GEF B DKO (DKO) mice. (Middle) Cell numbers of IgM^low^ or IgM^high^, B220^low^ immature B cells, IgM^low^, B220^high^ mature B cells in the bone marrow (*n* = 5). *^1^*P* < 0.001, *^2^*P* < 0.001, *^3^*P* < 0.001 versus corresponding WT cells. (Bottom) Representative CXCR4 and VLA-4 profiles of B220^+^ bone marrow cells. **b** (Top) Representative B220 and IgM profiles of B220^+^-gated blood cells from WT or DKO mice. (Middle) Representative IgM and IgD profiles of B220^+^-gated blood cells. (Bottom) Cell numbers of IgM^high^, IgD^low^ immature and IgM^low^, IgD ^high^ mature B cells from B220^+^-gated blood cells (*n* = 5). *^1^*P* < 0.001, *^2^*P* < 0.001 versus corresponding WT cells. **c** (Top) Representative IgM and CD43 profiles of B220^low^, IgM^−^-gated cells (progenitor B cells) from the bone marrow of WT or DKO mice. (Bottom) Cell numbers of IgM^−^, CD43^low^ pre-B and IgM^−^, CD43 ^high^ pro-B cells from B220^low^, IgM^−^-gated bone marrow cells (*n* = 5). *P < 0.001 versus corresponding WT cells. **d** (Top) Representative CD21 and CD23 profiles of B220^+^-gated spleen cells from WT or DKO mice. (Bottom) Cell numbers of CD21^−^, CD23^−^ immature, CD21^high^, CD23 ^low^ marginal zone (MZB) and CD21^high^, CD23 ^high^ follicular (FOB) B cells from B220^+^-gated spleen cells (*n* = 3). *^1^*P* < 0.001, *^2^*P* < 0.001 versus corresponding WT cells. **e** (Left) Lysates from WT or DKO B cells stimulated with CXCL12 at the indicated times were subjected to the pull-down assay. Bound Rap1 (Rap1-GTP) and total Rap1 were detected with anti-Rap1. (Right upper) Displacement of WT or DKO B cells was measured on VCAM-1 with or without CXCL12 (*n* = 30). (Lower) Representative tracks of WT or DKO B cells with CXCL12 are shown. Each line represents a single-cell track. Each bar graph represents the means ± SEM

In contrast to T cells, immature B cell egress from the bone marrow (BM) is a passive process, independent of pertussis toxin (PTX)-sensitive GPCR signaling such as S1P. On the other hand, the retention of these cells in the BM strictly depends on amoeboid motility mediated by CXCR4 and VLA-4 [25]. We also obtained the *Mb-1-cre/Rapgef2*^*f/f*^*/Rapgef6*-null mice (RA-GEF B DKO). As shown in Fig. 4a, the number of IgM^high^ and B220^low^ immature B cells in the BM of RA-GEF B DKO mice was reduced to 12% of the number in WT mice. In contrast, the number of immature B cells in the blood and spleen of RA-GEF B DKO mice increased approximately twofold compared to those of WT mice (Fig. 4b, d). The number of mature B cells (IgM^low^, B220^high^) in the BM of RA-GEF B DKO mice was also reduced to 11% of WT mice (Fig. 4a). Pre-B cells (IgM^−^ and CD43^low^), which differentiation was dependent on the interaction with stroma cells [26], were lower in the BM of RA-GEF B DKO mice, compared to WT mice (Fig. 4c). The expression of CXCR4 and VLA-4 in the BM B220^+^ cells of the RA-GEF B DKO mice was similar to those of the WT mice (Fig. 4a). These data suggest that the retention of immature B cells and the retention/recirculation of mature B cells in the BM depend on RA-GEFs.

Furthermore, marginal zone B cells (CD21^high^, CD23^lo^), which were reported to differentiate in an integrin-dependent manner, were significantly diminished in the spleen of RA-GEF B DKO mice (Fig. 4d). Consistent with those data, the Rap1-GTP levels in RA-GEF-deficient B cells were reduced to 16% of the B cell level derived from the control littermate mice at the peak point in time after CXCL12 stimulation. And, CXCL12-dependent migration on the VCAM-1-coated plate was significantly reduced in the RA-GEF-deficient B cells (Fig. 4e). Thus, RA-GEF plays crucial roles in chemokine and integrin-mediated motility that are critical for B cell maturation in the BM and the spleen.

### PLD2 is critical for T cell migration in response to CCL21

**Fig. 5:**
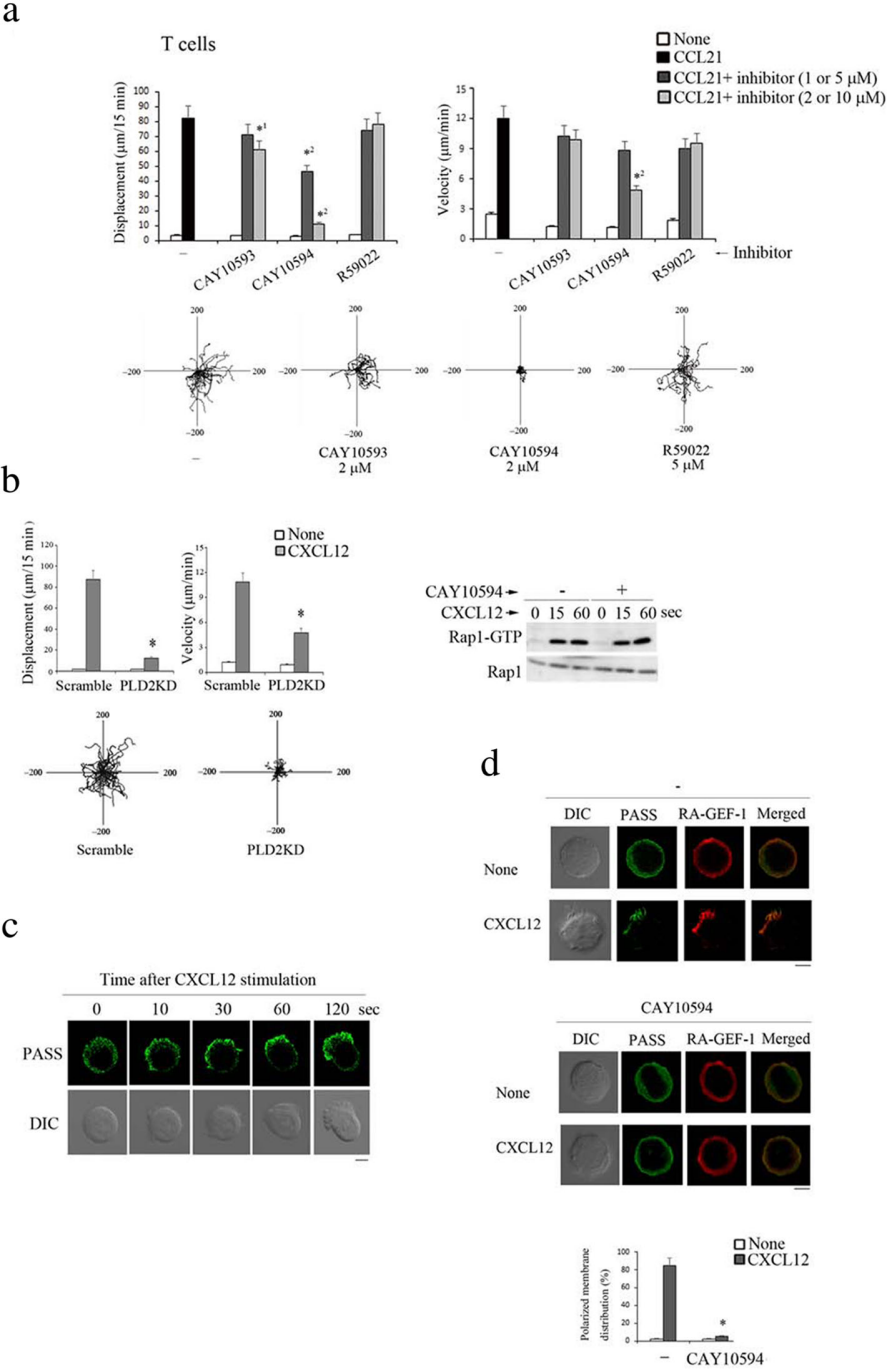
PLD2 is critical for chemokine-dependent PA generation at the plasma membrane. **a** (Top) Displacement (left) and velocity (right) of T cells were measured on ICAM-1 with or without CCL21 in the presence or absence of 1 or 2 μM of CAY10593, CAY10594, and 5 or 10 μM of R59022 (*n* = 30). *^1^*P* < 0.002, *^2^*P* < 0.001 versus WT T cells. (Bottom) Representative tracks of T cells treated with the indicated inhibitors with CCL21 are shown. Each line represents a single-cell track. **b** (Left upper) Displacement and velocity of scramble (control) or PLD2-knockdown cells were measured on the ICAM-1 with or without CXCL12 (*n* = 30). *P < 0.001 versus control cells. (Left lower) Representative tracks of control or PLD2 KD cells with CXCL12 are shown. Each line represents a single-cell track. (Right) BAF cells treated with or without CAY10594 were stimulated with CXCL12 at the indicated times, lysed, and subjected to the pull-down assay. Bound Rap1 (Rap1-GTP) and total Rap1 were detected with anti-Rap1. **c** PASS-GFP-expressing control cells were stimulated with CXCL12 for the indicated times. Time 0 represents the first time-lapse image; subsequent images were obtained in the same focal plane. Scale bar, 5 μm. **d** Distribution of PASS-GFP and RA-GEF-1 in BAF cells that were untreated (none) or treated with CXCL12 for 10 min in the presence (middle) or absence (top) of CAY10594 is shown. Scale bar, 5 μm. (Bottom) The graph shows the percentages of cells with the polarized membrane localization of PASS at the plasma membrane (*n* = 30). *P < 0.001 versus CXCL12-stimulated cells in the absence of CAY10594. Each bar graph represents the means ± SEM

Since RA-GEF plays essential roles in chemokine and S1P-mediated Rap1 activation and trafficking, we investigated the regulatory mechanisms of RA-GEF. Previous studies demonstrated that RA-GEF-1 interacts with PA [16]. Recently, we reported that a PLD inhibitor impaired CXCL12-dependent migration using a pro-B cell line referred to as Ba/F3 cells (BAF) [21]. In this study, we examined whether the PLD-dependent PA generation is involved in the promotion of primary T cell migration in response to CCL21. The inhibition of PA production in murine T cells by a PLD2-specific inhibitor (CAY10594), but not a PLD1-specific inhibitor (CAY10593), reduced CCL21-induced migration on the ICAM-1 (Fig. 5a). Treatment of T cells with R59022, a diacylglycerol kinase (DGK) inhibitor, did not affect CCL21-induced migration on the ICAM-1 (Fig. 5a). These data suggest that PA production from the hydrolysis of phosphatidylcholine by PLD2, but not the phosphorylation of DG via the DGK, plays a significant role in T cell migration downstream of the chemotactic factors. Next, using lentiviral transduction of short hairpin RNAs specific to PLD2, we knocked it down in BAF cells (PLD2 KD cells), in which the PLD2 expression was reduced to 29% of the control cells. Consistent with the results of T cells using CAY10594, PLD2 KD cells demonstrated defective migration upon stimulation with CXCL12 (Fig. 5b). However, CAY10594 did not inhibit Rap1 activation after stimulation with CXCL12 (Fig. 5b). These data indicated that PLD2-dependent PA generation was critical for chemokine-dependent cell migration, but not involved in the increase of GEF activity in RA-GEF for Rap1.

Our previous study also showed that the RA-GEF-1 mutant, which does not bind with PA but has intact GEF activity for Rap1, did not translocate to the plasma membrane, nor did it migrate on the ICAM-1 after chemokine stimulation [21]. This result suggested that PA was critical for membrane localization of RA-GEF-1 in integrin-mediated migration. Therefore, we examined the spatiotemporal process of PA generation using PASS as a PA reporter and also examined the recruitment of RA-GEF-1 to the PA-accumulated region of the plasma membrane in response to CXCL12. As shown in Fig. 5c, PASS was distributed diffusely in the cytoplasm of unstimulated cells and was quickly translocated to the plasma membrane and accumulated at the restricted region after CXCL12 stimulation, indicating that the chemokine induced the PA-rich membrane compartment within several minutes. The RA-GEF-1 was co-localized with the PASS on the plasma membrane of CXCL12-stimulated cells, which in turn was prevented by the CAY10594 (Fig. 5d). These data demonstrated that PLD2-dependent PA generation at the plasma membrane defined the membrane distribution of the RA-GEF-1 and played an indispensable role in T cell migration.

### The PA-dependent localization of RA-GEF determines the direction of T cell migration through the spatial distribution of Rap1-GTP

**Fig. 6:**
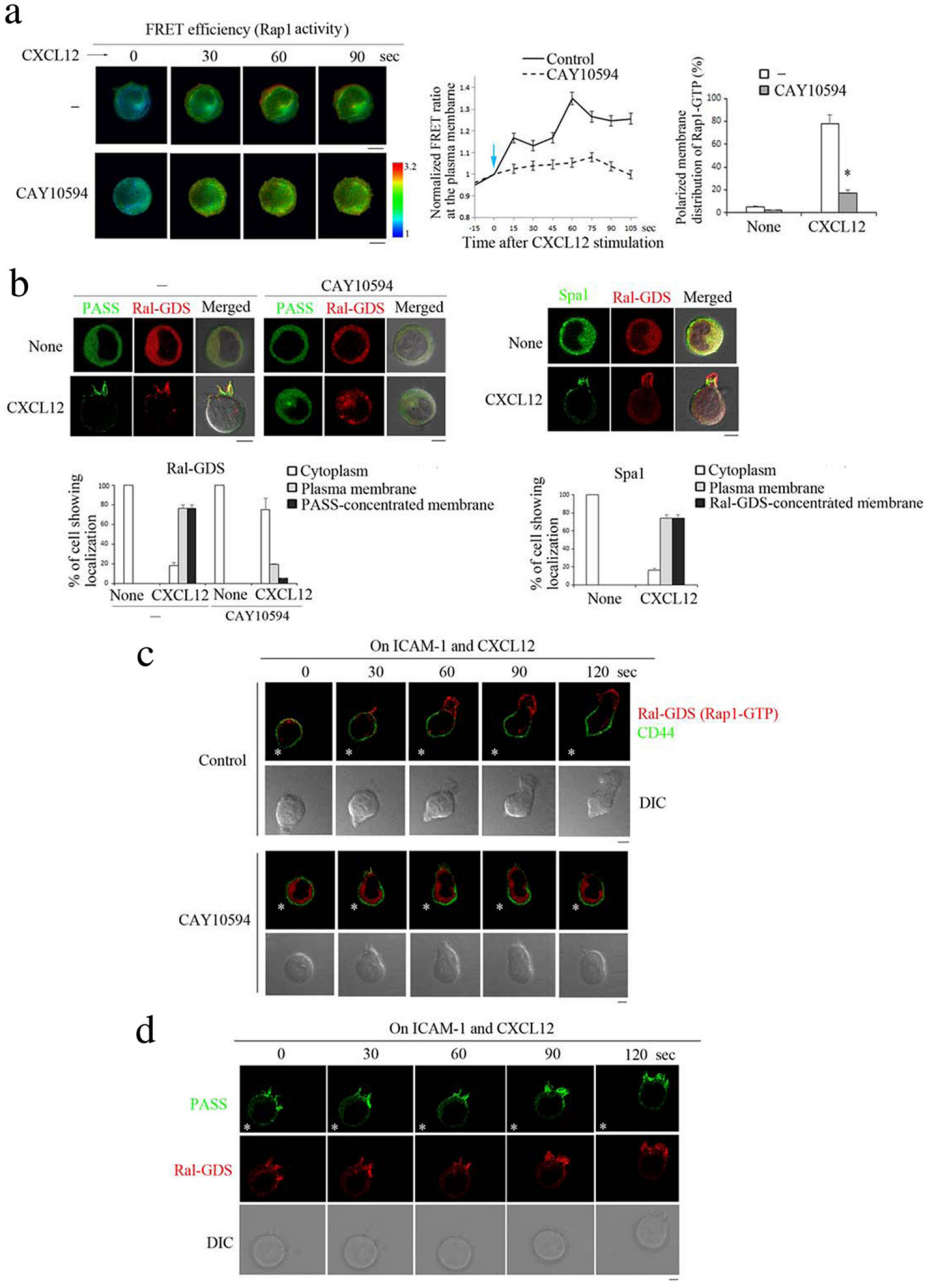
PA-dependent Rap1-GTP localization at the plasma membrane induces the development of the front membrane. **a** (Left) The FRET-based Rap1 activity sensor-expressing BAF cells were stimulated with CXCL12 in the presence or absence of CAY10594. An image of mTurquoise/Venus ratio represents FRET efficiency. (Center) The FRET efficiency at 6 μm edge region of plasma membrane is shown after CXCL12 stimulation (*n =* 20). Values are normalized to the level at zero point. The blue arrow marks the addition of CXCL12. (Right) The percentages of cells showing more than 1.3-fold increase in the FRET efficiency at the plasma membrane at 120 s after CXCL12 stimulation are shown (*n =* 20). *P < 0.001 versus CXCL12-stimulated cells without CAY10594. **b** (Left upper) Co-localization of PASS-GFP (PA) and Ral-GDS-RBD-mCherry (Rap1-GTP) in BAF cells at 10 min after CXCL12 stimulation with or without CAY10594 is shown. (Lower) We measured the ratios of Ral-GDS localized in the cytoplasm, plasma membrane, and PASS-concentrated region of plasma membrane of the cells. The graph shows percentages of cells showing that more than 50% of Ral-GDS was localized in each region (*n* = 30). (Right upper) Localization of Spa1-GFP and Ral-GDS-RBD-mCherry in BAF cells treated with CXCL12 is shown. (Lower) We measured the ratios of Spa1 localized in the cytoplasm, plasma membrane, and Ral-GDS-concentrated region of plasma membrane of the cells. The graph shows percentages of cells showing that more than 50% of Spa1 was localized in each region (*n* = 30). **c** Distribution of the Ral-GDS-RBD-mCherry and FITC-conjugated anti-CD44 in BAF cells on CXCL12 and ICAM-1-coated surface with (lower) or without (upper) CAY10594 is shown. Time 0 represents the first time-lapse image; subsequent images were obtained in the same focal plane. The asterisk symbol (*) shows the certain position. **d** Localization of PASS-GFP and Ral-GDS-RBD-mCherry in BAF cells on CXCL12 and ICAM-1-coated surface is shown. Each bar graph represents the means ± SEM. Scale bar, 5 μm

To explore whether the PA-dependent distribution of RA-GEF influences the temporal and spatial localization of Rap1 activity, we examined the effects of a PLD2 inhibitor on the distribution of Rap1-GTP using Förster resonance energy transfer (FRET) with the improved Rap1 activity sensor. Rap1 was present in the membrane fractions such as vesicles, plasma membranes, and endosomal compartments of the perinuclear region of unstimulated cells, and the distribution of Rap1 was unchanged after chemokine stimulation [2, 4, 27]. As previously reported [6], within 1 min, Rap1 activation occurred at the plasma membrane and the perinuclear region of the control cells. However, inhibition of the PA formation by the CAY10594 impaired activation of the Rap1 at the polarized region of the plasma membrane (Fig. 6a). This was similar to the defective distribution of RA-GEF [21] (Fig. 5d). The results suggest that PA-dependent translocation of RA-GEF to the plasma membrane is critical for the localization of Rap1-GTP.

Using a Ral guanine nucleotide dissociation inhibitor (GDS)-RBD (Ras-binding domain)-mCherry (Ral-GDS-RBD) as a Rap1-GTP reporter, the PASS was co-localized with Rap1-GTP in the plasma membrane of CXCL12-stimulated cells, and this region developed to the protrusion (Fig. 6b and Additional file 3: Figure S3A). CAY10594 prevented the accumulation and co-localization of PASS with Rap1-GTP at the plasma membrane and impaired the formation of the protrusion (Fig. 6b). This result suggests that PA promotes the formation of the front adherent membrane in Rap1-GTP-dependent manner.

Previous studies demonstrated that GTPase-activating protein (GAP) for Rap1 spatially regulates Rap1 activation, which is critical for chemotaxis [28]. We also observed the cellular localization of Spa1, a Rap1GAP. Spa1 was diffusely distributed in the cytoplasm of unstimulated cells and translocated to the plasma membrane after chemokine stimulation. The Spa1 accumulated in the proximal or overlapped region to Rap1-GTP-concentrated protrusion (Fig. 6b and Additional file 3: Figure S3A). This result showed that Spa1 was not excluded, but rather integrated into the PA-rich region of the plasma membrane by binding to the Rap1-GTP.

Because C3G is involved in lymphocyte trafficking, we examined the effect of dasatinib, an inhibitor for abl tyrosine kinase reported to be critical for C3G activation [13, 29, 30]. The dasatinib did not influence the accumulation and co-localization of the PASS and Rap1-GTP at the plasma membrane (Additional file 3: Figure S3B). At the same time, the knockdown of C3G did not affect cell migration or the localization of Rap1-GTP at the PA-concentrated region of the plasma membrane (Additional file 3: Figure S3C). However, the CXCL12-induced Rap1-GTP in the C3G KD cells was reduced by approximately 20% (Additional file 3: Figure S3D). These data showed that C3G was dispensable for migration and PA-dependent membrane localization of the Rap1-GTP.

We previously reported that the Rap1-GTP-concentrated protrusion is the adherent point on the integrin ligand [6]. We examined whether the PA-dependent Rap1-GTP membrane localization influenced cell movement on the ICAM-1. During cell migration on the plates coated with ICAM-1 and CXCL12, the Rap1-GTP-concentrated region on the plasma membrane extruded the protrusion, and typically developed to the front membrane, opposite the CD44-concentrated uropod. The cell moved along a front-rear axis (Fig. 6c). Similar to the results using the improved Rap1 activity sensor (Fig. 6a), the Rap1-GTP distributed diffusely over the CAY10594-treated cells (Fig. 6c). Defective development of the front membrane impaired cell locomotion (Fig. 6c). As expected, the PA and Rap1-GTP-concentrated membrane were developed to the front membrane during cell migration on the plates coated with ICAM-1 and CXCL12 (Fig. 6d). These data suggest that the PA determines subcellular localization of Rap1 activation and the direction of T cell movement.

### De-phosphorylation of RA-GEF is necessary for chemokine-dependent Rap1 activation

**Fig. 7:**
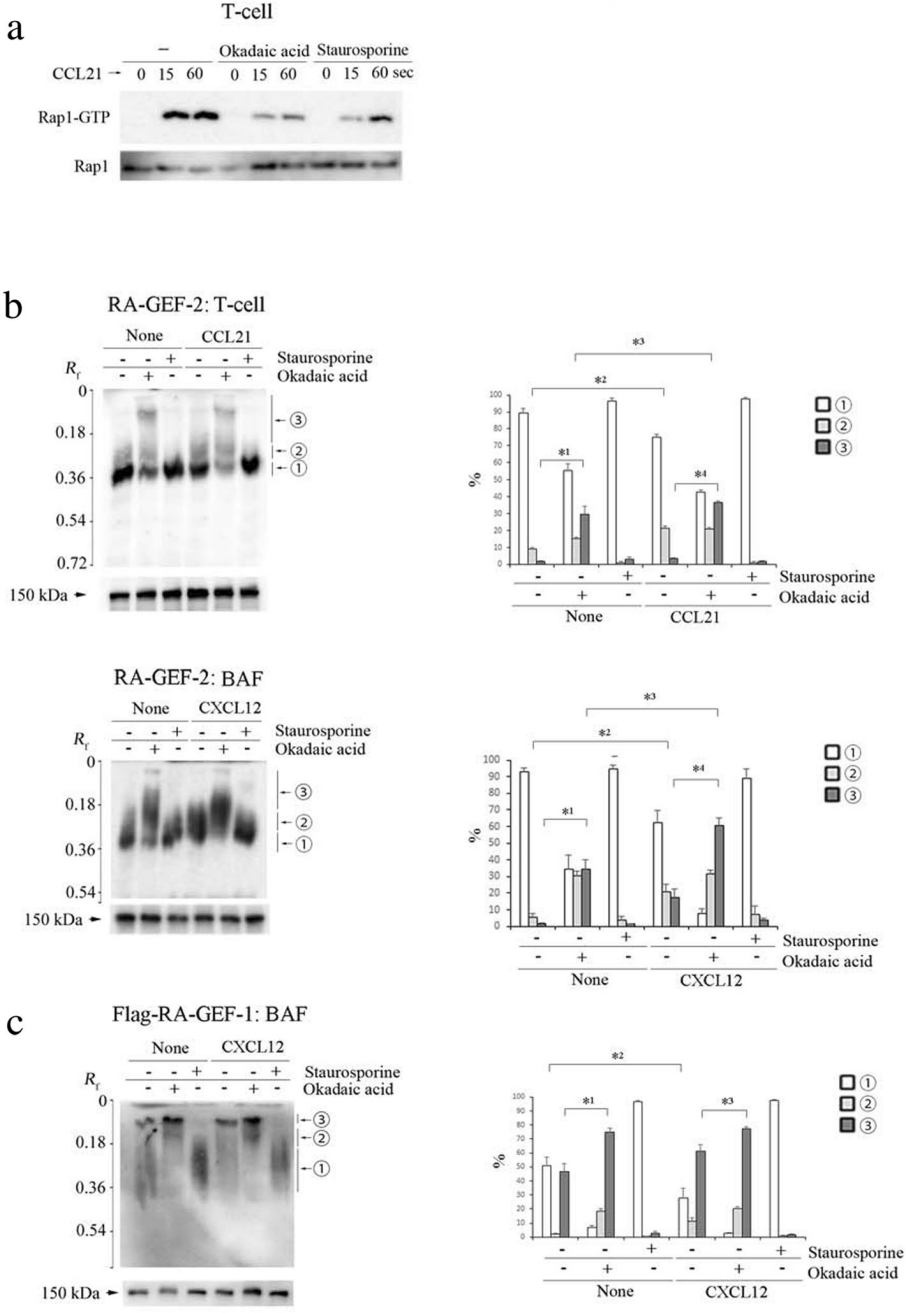
The de-phosphorylation of RA-GEF is necessary for Rap1 activation. **a** Mouse T cells untreated or treated with okadaic acid (OA) or staurosporine were stimulated with CCL21 and subjected to a pull-down assay. Bound Rap1 (Rap1-GTP) and total Rap1 were detected with anti-Rap1. **b** (Upper left) The de-phosphorylation of RA-GEF-2 in T cells stimulated with or without CCL21 for 60 s in the presence or absence of OA or staurosporine was analyzed by Phos-tag (upper) or conventional (lower) SDS-PAGE followed by immunoblotting with anti-RA-GEF-2. (Right) Quantification of ①, ②, and ③ bands, which is presented as percentage of each band. *^1^*P* < 0.006 versus unstimulated cells without OA, *^2^*P* < 0.01 versus unstimulated cells without OA, *^3^*P* < 0.05 versus unstimulated cells with OA, *^4^*P* < 0.001 versus CCL21-stimulated cells without OA. (Lower left) The de-phosphorylation of RA-GEF-2 in BAF cells stimulated with or without CXCL12 in the presence or absence of OA or staurosporine was analyzed by Phos-tag (upper) or conventional (lower) SDS-PAGE followed by immunoblotting with anti-RA-GEF-2. (Right) Quantification of ①, ②, and ③ bands, which is presented as percentage of each band. *^1^*P* < 0.005 versus unstimulated cells without OA, *^2^*P* < 0.04 versus unstimulated cells without okadaic acid, *^3^*P* < 0.008 versus unstimulated cells with OA, *^4^*P* < 0.004 versus CXCL12-stimulated cells without OA. **C** (Left)The de-phosphorylation of flag-RA-GEF-1 in BAF cells stimulated with or without CXCL12 in the presence or absence of OA or staurosporine was analyzed by Phos-tag (upper) or conventional (lower) SDS-PAGE followed by immunoblotting with anti-flag. (Right) Quantification of ①, ②, and ③ bands, which is presented as percentage of each band. *^1^*P* < 0.02 versus unstimulated cells without OA, *^2^*P* < 0.03 versus unstimulated cells without OA, *^3^*P* < 0.04 versus CCL21-stimulated cells without OA. The *R*_*f*_ value of 1.0 is defined as the position of the BPB dye. A representative of three independent experiments is shown. Each bar graph represents the means ± SEM

Because the association of RA-GEF with PA was not involved in the increased GEF activity for Rap1, we investigated the mechanisms for activating RA-GEF. As previously reported [31, 32], inhibition of protein kinases by staurosporine reduced chemokine-dependent Rap1 activation in primary T cells (Fig. 7a). Unexpectedly, okadaic acid (OA), an inhibitor of protein phosphatase 2 (PP2A), also suppressed the CCL21-induced Rap1 activation to 21% of the control T cell level (Fig. 7a). In addition, OA inhibited CCL21-dependent migration of primary T cells (Additional file 4: Figure S4A). Therefore, we explored the phosphorylation states of RA-GEF in primary T cells using a Phos-tag SDS-PAGE method. This method detected increases in phosphoprotein mobility and was capable of separating phosphoprotein from non-phosphoprotein. Because there is no useful antibody to detect endogenous RA-GEF-1 in a Phos-tag SDS-PAGE, we examined the phosphorylation states of RA-GEF-2 in primary T cells. Using Phos-tag SDS-PAGE followed by immunoblotting with an anti-RA-GEF-2 antibody, more than 80% of RA-GEF-2 was detected as a single band (①) in unstimulated primary T cells (Fig. 7b). In the presence of the OA, phosphorylated bands (②, ③) increased to 40–50% of the whole with or without CCL21 (Fig. 7b), indicating that RA-GEF-2 was basally phosphorylated but de-phosphorylated at the same time. CCL21 stimulation significantly increased the phosphorylated band (②), which was inhibited by staurosporine (Fig. 7b). In the Phos-tag free-condition, RA-GEF-2 was detected as a single band in the presence of the OA (Fig. 7b).

We also examined the phosphorylation states of RA-GEF-2 in BAF cells. RA-GEF-2 of unstimulated BAF cells was detected as a single band (①) in the Phos-tag SDS-PAGE (Fig. 7b). In the presence of the OA, phosphorylated bands (②, ③) increased to 60–80% of the whole with or without CXCL12 (Fig. 7b). These results indicated that RA-GEF-2 was constitutively phosphorylated but constantly de-phosphorylated in BAF cells as well as T cells. CXCL12 stimulation significantly increased the phosphorylated band (②, ③), which was inhibited by staurosporine (Fig. 7b). In the Phos-tag free-condition, RA-GEF-2 was detected as a single band in the presence of the OA (Fig. 7b).

We also investigated the phosphorylation states of flag-tagged RA-GEF-1, which was introduced into the BAF cell. Approximately 40% of RA-GEF-1 was basally phosphorylated in the unstimulated cells (③) (Fig. 7c). In the cells treated with staurosporine, RA-GEF-1 was detected primarily as a lower unphosphorylated band (①) (Fig. 7c). In the presence of OA, the most of RA-GEF-1 was observed as the phosphorylated bands (②, ③) in the presence or absence of CXCL12 (Fig. 7c). These results indicated that RA-GEF-1 were basally subject to phosphorylation and de-phosphorylation as well as RA-GEF-2. However, the phosphorylation of RA-GEF-1 dominated the de-phosphorylation in BAF cells, indicating that RA-GEF-1 was highly phosphorylated more than RA-GEF-2. CXCL12 stimulation induced the phosphorylation of RA-GEF-1 (②, ③) (Fig. 7c). In the Phos-tag free-condition, the RA-GEF-1 was detected as a single band (Fig. 7c).

Taken together, these results suggest that basal phosphorylation of both RA-GEF-2 and RA-GEF-1 suppresses chemokine-induced Rap1 activation.

**Fig. 8:**
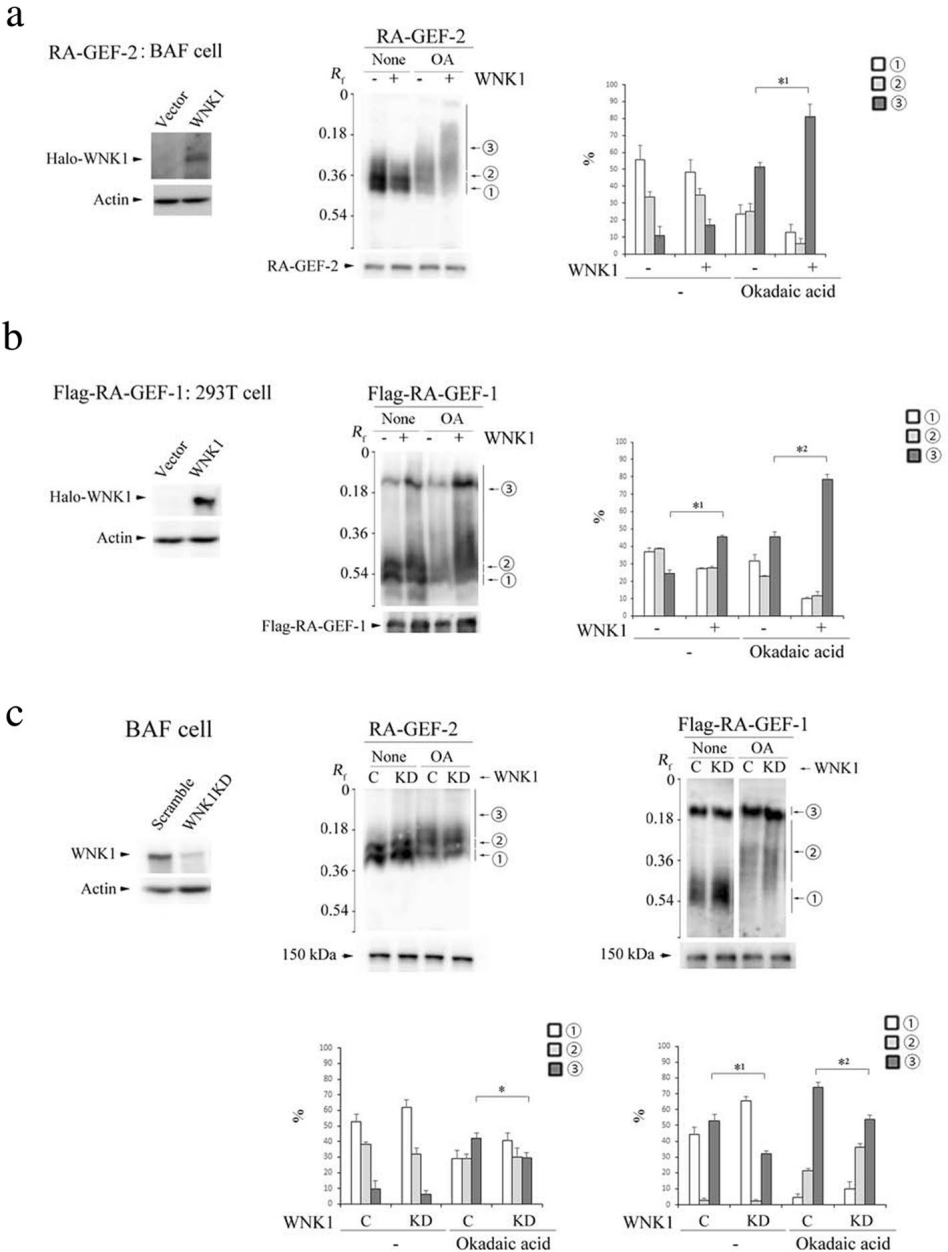
RA-GEF was basally phosphorylated by WNK1. **a** (Left) Total halo-WNK1 was detected with anti-halo. Actin was a loading control. (Middle) Control or WNK1-expressing BAF cells with or without OA were analyzed by Phos-tag (upper) or conventional (lower) SDS-PAGE followed by immunoblotting with anti-RA-GEF-2. (Right) Quantification of ①, ②, and ③ bands, which is presented as percentage of each band. *^1^*P* < 0.01 versus control cells with OA. **b** (Left) Total halo-WNK1 was detected with anti-halo. Actin was a loading control. (Middle) Flag-RA-GEF-1, and control or WNK1-expressing 293T cells with or without OA were analyzed by Phos-tag (upper) or conventional (lower) SDS-PAGE followed by immunoblotting with anti-flag. (Right) Quantification of ①, ②, and ③ bands, which is presented as percentage of each band. *^1^*P* < 0.002, *^2^*P* < 0.002 versus control cells in the presence or absence of OA. **c** (Left) Total WNK1 was detected with anti-WNK1. Actin was a loading control. (Middle top) Control or WNK1-knockdown (KD) BAF cells with or without OA were analyzed by Phos-tag (upper) or conventional (lower) SDS-PAGE followed by immunoblotting with anti-RA-GEF-2. (Bottom) Quantification of the abundance of ①, ②, and ③ bands, which is presented as percentage of each band. *P < 0.05 versus control cells in the presence of OA. (Right top) Control or WNK1KD, flag-RA-GEF-1-expressing BAF cells with or without OA were analyzed by Phos-tag (upper) or conventional (lower) SDS-PAGE followed by immunoblotting with anti-flag. The Phos-tag blot of flag-RA-GEF-1 was cropped from a same blot. (Bottom) Quantification of ①, ②, and ③ bands, which is presented as percentage of each band. *^1^*P* < 0.009, *^2^*P* < 0.008 versus control cells in the absence or presence of OA. The *R*_*f*_ value of 1.0 is defined as the position of the BPB dye. A representative of three independent experiments is shown. Each bar graph represents the means ± SEM

Previous study [33] reported that knockdown of lysine deficient protein kinase 1 (WNK1) in Jurkat T cells augmented CXCL12-dependent Rap1 activation, indicating that WNK1 suppressed Rap1 activation in T cells. As shown in Additional file 4: Figure S4B, the overexpression of WNK1 in BAF cells reduced Rap1 activation in response to CXCL12, so we examined the involvement of WNK1 in basal phosphorylation of endogenous RA-GEF-2 in BAF cells. The overexpression of WNK1 significantly promoted the phosphorylation of RA-GEF-2 in the presence of OA (Fig. 8a and Additional file 4: S4C). However, without OA, the overexpression of WNK1 slightly increased the phosphorylation of the RA-GEF-2. This suggests that PP2A actively de-phosphorylates the residue(s) of RA-GEF-2 phosphorylated by the WNK1.

Because the flag-RA-GEF-1 did not basally undergo intense phosphorylation in 293T cells, we also examined the effect of WNK1 on the phosphorylation of flag-RA-GEF-1 using the 293T cells. The overexpression of WNK1 virtually promoted the phosphorylation of flag-RA-GEF-1 in the presence or absence of OA (Fig. 8b). Thus, both the RA-GEF-1 and RA-GEF-2 were possible substrates of WNK1.

Finally, in the absence or presence of OA, we examined the effects of the knockdown of WNK1 on basal phosphorylation of RA-GEF-2 and flag-RA-GEF-1 in BAF cells (Fig. 8c). The knockdown of WNK1 increased the CXCL12-dependent Rap1 activation (Additional file 4: Figure S4D). As shown in Fig. 8c, in the presence of OA, the knockdown of WNK1 significantly decreased the upper phosphorylated band (③) and instead increased the unphosphorylated band (①) of RA-GEF-2. On the other hand, in the absence or presence of OA, the knockdown of WNK1 increased the unphosphorylated band (①) or phosphorylated band (②) of the flag-RA-GEF-1 (Fig. 8c). These data indicated that basal phosphorylation of RA-GEF-2 and RA-GEF-1 was partially dependent on WNK1. In addition, we confirmed that the knockdown of WNK1 did not affect the translocation of RA-GEF-1 to the plasma membrane (Additional file 4: Figure S4E).

**Fig. 9:**
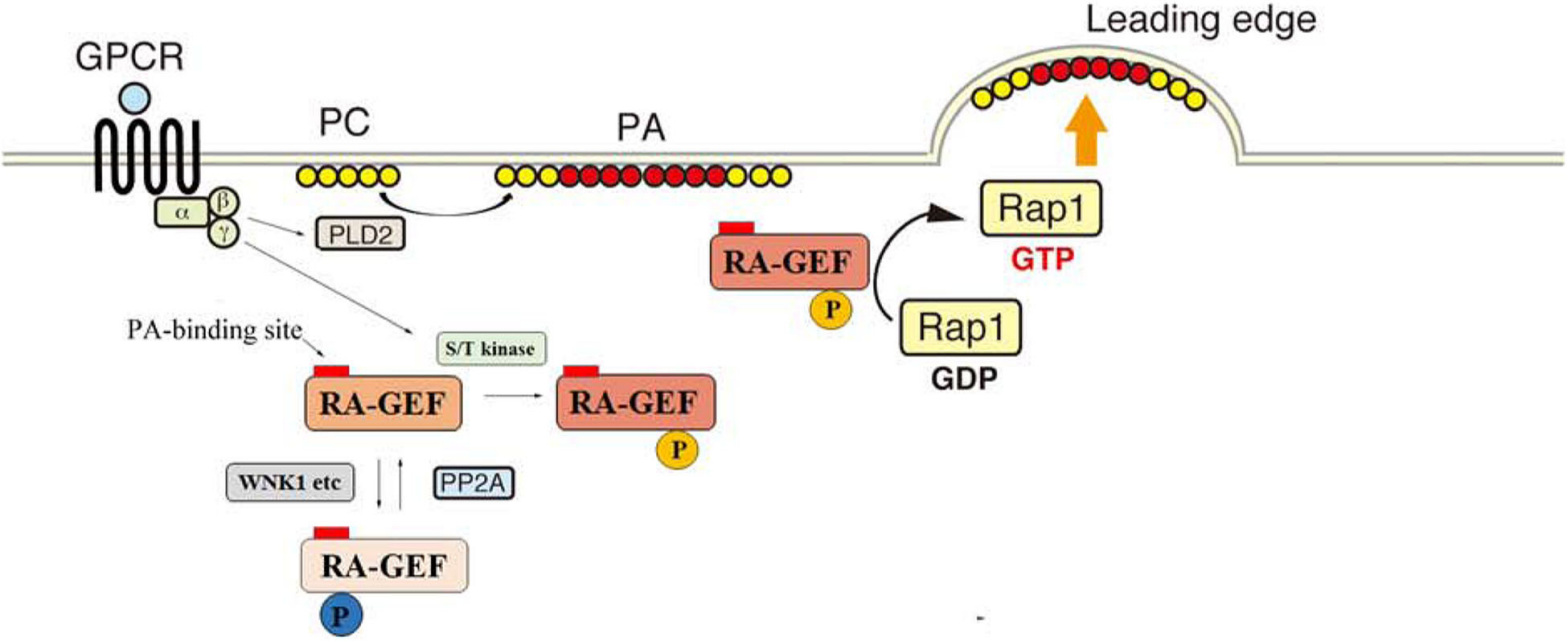
Model for RA-GEF regulation in T cells. In unstimulated cells, RA-GEF is distributed at the cytoplasm, and their phosphorylation/de-phosphorylation at similar residue(s) is co-occurring by WNK1 and other kinases, and PP2A. Gαi protein-coupled receptor (GPCR)-mediated signaling produces PA at the plasma membrane via PLD2, and RA-GEF translocates to the PA-generated region of plasma membrane. GPCR-mediated signaling phosphorylates RA-GEF at different residue(s), which activates RA-GEF. Activated RA-GEF converts Rap1-GDP to Rap1-GTP at PA-concentrated region of the plasma membrane and induces the protrusion, which develops the front membrane

Taken together, these results suggest that Rap1 activation by chemotactic factors requires basal de-phosphorylation of RA-GEF by PP2A, because their GEF activity might be suppressed by the phosphorylation by protein kinases such as WNK1 (Fig. 9).

## Discussion

To understand the regulation of lymphocyte trafficking, it is important to identify the Rap1-GEFs involved in chemokine and S1P-mediated signaling. This study shows that RA-GEF-1/2 are major GEFs involved in chemokine-dependent Rap1 activation of both T and B cells. In T cells, Rap1 is activated by S1P through RA-GEF-1/2 (RA-GEF) and is essential for thymic egress. Since thymic egress is reduced in T cell-specific *Rap1a/b*-double knockout mice, we conclude that RA-GEF-dependent Rap1 activation plays indispensable roles in both T cell homing and egress. On the other hand, RA-GEF plays a critical role in the retention of B cells in the BM; thereby, their deficiency promotes egress from the BM. In contrast to T cells, B cell numbers in peripheral LNs of mice having B cell-specific knockouts of *Rapgef2/6* were not significantly reduced. This indicated that RA-GEF was dispensable for the homing of B cells into LNs. Because Rap1a/b deficiency inhibits the homing of both T and B cells into LNs, the remaining Rap1 activity of RA-GEF B DKO mice was enough for B cells to adhere and transmigrate across the high endothelial cells (HEV). Approximately 20% of Rap1 activation remained in the RA-GEF-deficient T and B cells via GPCR-mediated signaling. CalDAG-GEFI is not expressed in mouse naïve lymphocytes [34]. As C3G is another major Rap1-GEF expressed in lymphocytes, in RA-GEF-deficient lymphocytes, C3G may compensate for chemokine-dependent Rap1 activation [13–15].

Because lymphocytes are non-adherent cells, integrin on the cell surface of lymphocyte must be activated to migrate on the integrin ligand. We previously reported that the conversion of Rap1-GDP to Rap1-GTP at the plasma membrane induced the integrin clustering which formed adhesion point at the leading edge, and facilitated the lymphocyte migration [4, 35, 36]. However, it is unclear how the cellular distribution of Rap1-GTP and the direction of cell movement are determined. We revealed the model for the mechanisms underlying the spatial and temporal regulation of Rap1 activation via RA-GEF downstream of chemotactic factors (Fig. 9). GPCR-dependent PA generation on the plasma membrane recruited RA-GEF, where they activated the Rap1. Consequently, the PA and Rap1-GTP-concentrated membrane extended a protrusion, developed the adhesive front membrane, and established cell polarization necessary for directional migration. On the other hand, the temporal activation of RA-GEF by chemotactic factors was regulated by phosphorylation and de-phosphorylation (Fig. 9). Both RA-GEF-1/2 are basally phosphorylated by WNK1 and other unknown kinases, but consistently de-phosphorylated by PP2A. Previous studies demonstrated the importance of RA-GEF phosphorylation for Rap1 activation [31, 32]. This study demonstrated that in response to chemokine, basal de-phosphorylation of RA-GEF was also necessary for Rap1 activation. Thus, these data suggest that localization and activation of RA-GEF are tightly regulated via PA and phosphorylation/de-phosphorylation, respectively.

The direct phosphorylation of human RapGEF2 at Ser960 by PKC-θ was involved in T cell receptor-mediated Rap1 activation [31]. Cdk5-dependent phosphorylation of Ser1124 was important for the activation of human RA-GEF-1 [32]. This study suggests that phosphorylation of RA-GEF is necessary for chemokine-dependent Rap1 activation. On the other hand, WNK1-deficient T cells demonstrated an augmented chemokine-dependent Rap1 activation [37], suggesting that phosphorylation by WNK1 inhibits Rap1 activation. In this study, we demonstrated that WNK1 phosphorylated RA-GEF, and basal de-phosphorylation of RA-GEF was critical for chemokine-dependent Rap1 activation in lymphocytes. These data suggest the existence of distinct phosphorylation sites in RA-GEF, which suppress or promote the GEF activity for Rap1.

In this study, Spa1 immediately moved to the Rap1-GTP-generating sites on the plasma membrane, suggesting that Rap1 on-off was rapidly regulated on the adhesion points by Spa1. Rap1-GTP-dependent development of a new adherent membrane may cause high-speed movement and active changes in the direction toward the chemotactic gradient as a driving device. Adherent lymphocytes partially move in ways similar to other adherent cells, but often change the direction of movement. In contrast to neutrophils that trace pathogens, lymphocytes interact and communicate with other cells such as lymphocytes or dendritic cells. So, random migration on chemokine-presented cells is suitable for lymphocytes to increase the opportunity of encountering other cells.

The accumulation of Qa-2^+^CD24^low^ SP in the thymus of RA-GEF-deficient mice was consistent with the defect observed by the inhibition of thymic egress with FTY720 [11]. CD4 SP and CD8 SP are located in the perivascular spaces surrounding large blood vessels at the corticomedullary junction and the medulla, and migrate from the thymus through blood vessels [8]. The regulation of S1P gradients between the thymus and circulatory fluids directs the chemotactic egress of mature thymocytes from the thymus into the circulatory system [7]. We originally developed the chamber forming the stable S1P gradient in vitro and demonstrated that RA-GEF-1/2 are important for the directional bias of T cells toward the S1P gradient. Thus, RA-GEF-dependent Rap1 activation was found to be crucial for S1P-mediated reverse transmigration across the endothelium through blood vessels at the corticomedullary junction.

Previously, our studies demonstrated that Mst1 was downstream effector molecule of Rap1-GTP and mediated cell polarization and migration [5]. Previous studies demonstrated that thymic egress is reduced in Mst1-deficient mice [5, 11]. In this study, chemokine or S1P-mediated Mst1 activation was impaired by an RA-GEF deficiency. Reduced Mst activation in RA-GEF-deficient T cells may cause impaired thymic egress.

Rap1 deficiency in T cells causes severe colitis with tubular adenoma [6]. The knockout of RAPL or Mst1 also leads to the development of autoimmune diseases [38, 39]. These findings demonstrate that lymphopenia preceding homeostatic T cell proliferation in the lymph nodes impairs self-tolerance. In this study, we found that RA-GEF-1/2 are key GEFs for Rap1 that determine the direction of motile lymphocytes. Therefore, it is important for the control of immune responses and tolerance to clarify the regulatory mechanisms of RA-GEF in T cells.

## Conclusions

RA-GEF-1 and 2 are major Rap1-GEFs in lymphocyte trafficking. Membrane distribution and activation of RA-GEF are key factors for a directional movement of lymphocytes. Phosphatidic acid determines spatial distribution of Rap1-GTP through the regulation of membrane localization of RA-GEF. The activation of RA-GEF with chemokine requires basal de-phosphorylation.

## Methods

### Mice

All animal experiments were carried out in accordance with the Regulations for the Care and Use of Laboratory Animals in Kitasato University, and the protocols used in this study were ethically approved by the Institutional Animal Care and Use Committee for Kitasato University. *Rapgef6*^−/−^ mice were generated as described previously [19]. *Rapgef2*^*flox/flox*^ mice were crossed with *Lck-Cre or Cd4-Cre mice*, *yielding mice with T cell-specific deletion of Rapgef2*. *Rapgef2 conditional knockout* (*Rapgef2*^*flox/flox*^: *Lck-Cre or Cd4-Cre*) and *Rapgef6*^*+*/*−*^ male mice were crossed with *Rapgef2*^*flox/flox*^, *Rapgef6*^*+*/*−*^ female mice, yielding *Rapgef2*^*lox/flox*^, *Rapgef6*^*−*/*−*^ mice. In all experiments, 7–10-week-old littermates (both male and female) were used. All experiments were performed in accordance with the protocols approved by the Animal Care and Use Committee of Kitasato University (Kanagawa, Japan).

### Cell lines

BAF-LFA1 cells (Ba/F3 cells expressing human LFA-1) have been described [40]. BAF-LFA1 cells have not been authenticated since fingerprint for Ba/F3 cells is not publicly available. All cell lines were tested for mycoplasma contamination by 4′,6-diamidino-2-phenylindole (DAPI) staining with negative results.

### Antibodies and reagents

Fluorescence-conjugated anti-mouse CD3, B220, CD4, CD8, CD62L, CD44, CD24, Qa-2, CD69, TCRβ, CD25, IgM, IgD, CD21, CD23, CD43, VLA-4, CXCR4, and CCR7 (e-bioscience); anti-mouse LFA-1; anti-C3G (Santa Cruz Biotechnology); anti-RFP (MBL); anti-Rap1 (BD Biosciences); pMst1/2; Mst1; PLD2 (Cell Signaling); anti-FLAG (Wako); anti-Halo (Promega); anti-WNK1 (R&D systems); β-actin; and peroxidase-conjugated goat anti-rat, rabbit, or mouse IgG (Cell Signaling) were used for flow cytometry (1:100) and immunoblotting (1:1000). Mouse CCL21, CCL19, and CXCL12 (R&D Systems) were used at the concentration of 100 nM for the assays. 0.5 μM staurosporine (protein kinase inhibitor) (Wako Pure Chemicals), 0.5 μM okadaic acid (PP2A inhibitor) (Wako), 5–10 μM R59022 (Tocris Bioscience) (DGK inhibitor), 1 μM dasatinib (abl family PTK inhibitor) (Carbosynth), and 1–2 μM CAY10594 (PLD2 inhibitor) or CAY10593 (PLD1 inhibitor) (Cayman Chemical) were used for examination of their roles in chemokine-dependent Rap1 regulation.

### RT-PCR

Total RNA was extracted from T and B cells with TRIzol reagent, and total RNA was purified according to the manufacturer’s protocol (Invitrogen). The RNA was transcribed into cDNA with Takara Prime script reverse transcriptase according to the manufacturer’s protocol (Takara). The sequence of the primers used was as follows: RA-GEF-1, forward 5′-aaggcgacacaggcactatc-3′ and reverse 5′-ccttctgggaaatcggcaat-3; RA-GEF-2, forward 5′-cggacacatgtgaaccaaac-3′ and reverse 5′-atccaatcttcgagggaacc-3′. The PCR products were loaded on 2% agarose gels.

### DNA constructs and transfection

The cDNA encoding mouse RA-GEF-1 was amplified by PCR using the following primers (restriction sites are underlined): forward 5′-ggaattcgaaatggctttccttgtgcg-3′ and reverse 5′-tagcggccgccaaacagcagacacttgttc-3′. The amplified cDNA was subcloned into a pcDNA-FLAG vector.

To produce a mammalian expression vector of mCherry-tagged Ral-GDS-RBD, we subcloned cDNAs encoding mCherry and the RBD of human Ral-GDS (amino acids 772–868) of pcDNA3.1 or a lentivirus vector (CSII-EF-MCS; a gift from H. Miyoshi, RIKEN, Wako, Japan). An improved FRET-based Rap1 activity sensor, consisting of an improved version of a yellow fluorescent protein (Venus)-tagged Rap1 linked with a monomeric Turquoise (mTurquoise)-tagged RAPL-RBD, was constructed into a pcDNA3.1 or a lentivirus vector (CSII-EF-MCS).

An enhanced green fluorescent protein-PA biosensor with superior sensitivity (EGFP-PASS) was generated as previously described [22]. A nuclear export sequence (NES) derived from protein kinase A inhibitor α (PKI-α) was added between EGFP and Spo20-PABD (PA-binding domain) cloned into pEGFP-C1. PASS tagged with monomeric GFP (mGFP) was generated by replacing EGFP in the EGFP-PASS with mGFP and mRFP. The lentiviral vectors carrying GFP-PASS were subcloned into pCDH-CMV-MCS (System Biosciences, Mountain View, CA). WNK1 cDNA and RA-GEF-2 transferred to a pFN21A vector were purchased from Kazusa. RA-GEF-2 cDNA was as subcloned into a pcDNA-flag vector. The fidelity of all constructs was verified by sequencing.

### RNA-mediated interference and gene introduction via lentiviral transduction

RNA-mediated interference was used to suppress mouse PLD2, C3G, and WNK1 expression. A 19-nucleotide-specific sense RNA sequence (GACACAAAGTCTTGATGAG) (5′–3′) or a scrambled control RNA sequence of PLD2, and GGGCTTTGGTGTTGAGTGT (5′–3′) or a scrambled control RNA sequence of C3G, and three kinds of specific sense RNA sequence GCTGCGTATTGAAGATATTAA (5′–3′), AGACGTTGCTTCTGGTATGA (5′–3′), and AAGATCTTGATGCTCAGTTGA (5′–3′) of WNK1 were introduced into BAF/LFA-1 using lentivirus with a lentivirus vector with or without GFP or puromycin resistance gene (a gift from Dr. Miyoshi H., RIKEN, Wako, Japan) containing the RNAi construct under control of the H1 promoter cassette, respectively. The production and concentration of lentivirus particles were assessed as previously described [35]. The transduction efficiencies were greater than 90%. A GFP high population was collected by cell sorting and subjected to adhesion assays and immunoblot analysis.

### Immunoprecipitation and immunoblot analysis

BAF cells or mouse T lymphocytes were lysed in a buffer (1% Nonidet P-40, 150 mM NaCl, 25 mM Tris-HCl [pH 7.4], 10% glycerol, 2 mM MgCl_2_, 1 mM phenylmethylsulfonyl fluoride, 1 mM leupeptin, and 0.1 mM aprotinin). Cell lysates were subjected to immunoblotting [2].

Phos-tag SDS-PAGE was performed according to the manufacturer’s protocol. Acrylamide gel 3.3% was mixed with 20 μM Phos-tag acrylamide (AAL-107, Wako Pure Chemicals) and 40 μM MnCl_2_. After sample separation, the gel was washed with blotting buffer containing 5 mM EDTA for 30 min to chelate Mn^2+^. Proteins were transferred onto PVDF membrane and used for immunoblotting.

### Pull-down assays

Rap1-GTP was pulled down with a glutathione *S*-transferase (GST)-RBD of RalGDS (1–149 of human Raf1) fusion protein, respectively [41]. Briefly, 10^7^ cells were lysed in ice-cold lysis buffer (1% Triton X-100, 50 mM Tris-HCl [pH 7.5], 100 mM NaCl, 10 mM MgCl_2_, 1 mM phenylmethylsulfonyl fluoride, 1 mM leupeptin, and 0.5 mM aprotinin) and incubated for 1 h at 4 °C with GST-fusion proteins coupled to glutathione sepharose beads. The beads were washed three times with lysis buffer and subjected to immunoblot analysis using an anti-Rap1 antibody. Immunoblotting of total cell lysates (5 × 10^4^ cells) was also performed.

### Lymphocyte migration on ICAM-1 and VCAM-1

Migration on ICAM-1 or VCAM-1 was performed as previously described using a ΔT dish (Bioptechs Inc.) with immobilized recombinant human or mouse ICAM-1Fc (0.5 μg/ml) or mouse VCAM-1Fc (0.5 μg/ml) [5, 36]. A total of 2 × 10^5^ cells were loaded onto the ICAM-1-coated dish. Phase-contrast images were obtained using an Olympus Plan Fluor DL × 10/0.3NA objective every 15 s for 10–15 min at 37 °C using a heated stage for ΔT dishes (Bioptechs Inc.). The frame-by-frame displacements and lymphocyte velocities were calculated by automatically tracking individual cells using the MetaMorph software (Molecular Devices). In each field, 30 randomly selected cells were manually tracked to measure the median velocity and displacement from the starting point.

### Thymic emigration

Thymic egress was measured using thymic lobes as described previously [10]. Thymic lobes isolated from WT and DKO mice without disrupting the capsule were incubated in the upper chamber of a Transwell (5-μm pore size) with CCL19 (100 nM) in the lower chamber. After 3 h, cells in the lower chamber were recovered and counted, and then immunostained with CD3, CD4, CD8, and CD62L. The cell numbers were calculated as the frequencies of the respective population.

### Transwell migration assay

Five hundred microliters of RPMI medium containing S1P (Sigma-Aldrich) was added to the lower chamber. One million thymocytes in 150 μl of medium were then added to the upper chamber of 3-μm pore 24-well tissue culture inserts and incubated for 3 h at 37 °C in a 5% CO_2_ incubator. The thymocytes from the input and lower chambers were counted and stained with CD3, CD4, CD8, and CD62L for flow cytometry. Percentages of migration were calculated for each flow cytometry-defined subset by dividing the number of that subset in the input with the number of migrated cells.

### Chamber fabrication and assay for chemotaxis toward S1P gradient using chambers

A micro-chamber for the chemotaxis assay was fabricated following a photolithography process described earlier [42, 43]. In brief, polydimethylsiloxane (PDMS; Sylgard 184 Silicone Elastomer Kit, Dow Corning) solution with a mixing ratio of 10:1 (base: curing agent) was poured on a 50-μm-thick SU8-mold (SU-83050; MicroChem) and was cured for 1 h at 75 °C. The PDMS sheet was then peeled off from the mold. Inlets for chemoattractant and cell-loading were opened with a 1.5-mm- or 2-mm-diameter biopsy punch (BP-15F, BP-20F; Kai industries), respectively. The fabricated PDMS was cut using a stainless steel corer (BSV01; TKG) to form a round 10-mm-diameter disc. A glass-bottom dish (P35G-0-14-C; MatTek) was treated with O_2_ plasma for 10 min to clean the glass surface using a plasma etcher (FA-1; Samco). The dish and the PDMS disc were treated with an O_2_ plasma for an additional 5 s, attached together by hands, and immediately heated on a hot plate for 3 min at 80 °C for permanent bonding.

Custom-made migration chambers were coated with mouse ICAM-1 Fc (0.5 μg/ml) and overlaid with 30 μl of 2 mM S1P with 10 μl of 0.4 μg/ml Alexa Fluor 594 dye (Thermo Fisher Scientific). The dimensions of the chamber are 500 μm wide, 50 μm high, and 1 mm long. Ten microliters of cell suspension (5 × 10^5^ cells) was casted into the chamber and observed at 37 °C for 180 min via time-lapse video microscopy. Cells were tracked using the MetaMorph software.

### Immunofluorescence staining

Cryostat sections of frozen tissues (10-μm thickness) were fixed with acetone, air-dried, and stained with the indicated antibodies. Chemokine-stimulated T lymphocytes were stained with the indicated antibodies as previously described [5]. Stained samples were observed with a confocal laser microscope (TCS SP8, Leica). Cells with segregated LFA-1 and CD44 accompanied with elongated cell shapes were considered polarized cells.

### Confocal microscopy and time-lapse imaging

Non-adherent cells incubated with or without chemokine were fixed in suspension and immobilized on poly-l-lysine-coated slides before staining. Confocal images (TCS, SP8, Leica) were obtained using a × 63 objective lens [35]. Time-lapse confocal images were also obtained in multitrack mode. Line profiles of the confocal images were obtained with ImagePro software (MediaCybernetics).

### Homing assay

Purified T cells were labeled with 1 μM 5,6-carboxyfluorescein diacetate (CFSE, Invitrogen) and 10 μM (5-(and-6)) ((4-chloromethyl) benzoyl) amino) tetramethylrhodamine) (CMTMR, Invitrogen). An equal number of labeled control and RA-GEF-deficient T cells (1–5 × 10^6^) were injected intravenously into a normal C57BL/6 mouse. After 1 h, inguinal and axillary LN cells and peripheral blood mononuclear cells were analyzed by flow cytometry [10]. Reversal of fluorescent dyes yielded the same results.

### Statistical analysis

Statistical analysis was performed using two-tailed Student’s *t* test. *P* values less than 0.05 were considered significant.

## Supplementary information

**Additional file 1: Figure S1.***Rapgef2* and *6* in T cells. (**A**) Expression analysis of *Rapgef2* and *6* in T and B cells by RT-PCR. RNA isolated from purified T cells and B cells was reverse transcribed and used as template for PCR amplification. PCR reactions were performed using *each* specific primer under optimal conditions. **(B**) Effect of *Rapgef2* or *6* deficiency on CCL21-Rap1 activation in T cells. GTP-bound Rap1 was analyzed by a pull-down assay using GST-RalGDS-RBD. RA-GEF-1 (right) or RA-GEF-2 (left)-deficient mouse T cells stimulated with 100 nM of CCL21 at the indicated times, lysed and subjected to a pull-down assay. Bound Rap1 and total Rap1 were detected by immunoblotting with an anti-Rap1 antibody.

**Additional file 2: Figure S2.** Gradient formation in the chemotaxis chamber. (**A**) A chamber overview. The chamber consists of a block of molded PDMS bonded to a glass-bottom dish that together form a 2 mm long linear channel. The height and the width of the channel are 50 μm and 250 μm, respectively. One side of the channel is connected to a cell-loading well, and the other side is connected to a chemoattractant circular reservoir of a diameter 2 mm. The reservoir is connected to an inlet for chemoattractant loading though a 200 μm-wide channel. The concentration gradient is formed by passive diffusion (green) by closing the inlet with a plug. Scale bar, 1 μm. (**B**) Snapshots of the concentration gradient in the chemotaxis chamber where a green food coloring was loaded for demonstration. Scale bar, 2 mm. (**C**) Evaluation of the gradient profile; PBS containing 10 μM fluorescein was loaded for visualization. The time-course of the fluorescent intensity profiles during the initial transient (left panel; immediately after loading) and after the initial transient (right panel; 30 min after loading). The mean fluorescence intensities of a 250 μm wide area as a function of the distance from the border between the channel and the cell-loading well; data was plotted every 5 min (left panel) and 10 min (right panel), respectively. (**D**) The time-course of the fluorescent intensity. The fluorescent intensity at 0 μm (dark blue), 500 μm (blue), 1000 μm (cyan), 1500 μm (yellow), and 1800 μm (red) from the cell-loading well during the initial transient (left panel; immediately after loading) and chemotaxis assay (right panel; 30 min after loading).

**Additional file 3: Figure S3.** Localization of PA-dependent Rap1-GTP in the front membrane during cell migration. (**A**) (Top left) We measured the ratios of Ral-GDS (red) in the cytoplasm, plasma membrane and PASS (green)-concentrated region of plasma membrane (white rectangular) in unstimulated and CXCL12-stimulated cells. (right) The graph shows ratios of Ral-GDS localized in each region (cytoplasm, plasma membrane or PASS-concentrated membrane). (Bottom left) We measured the ratios of Spa1 (green) in the cytoplasm, plasma membrane and Ral-GDS (red)-concentrated region of plasma membrane (white rectangular) in unstimulated and CXCL12-stimulated cells. (right) The graph shows ratios of Spa1 localized in each region. (**B)** (Left) Co-localization of PASS-GFP and Ral-GDS-RBD-mCherry in BAF cells after CXCL12 stimulation in the presence of dasatinib is shown. (Right) The ratios of Ral-GDS localized in the cytoplasm, plasma membrane and PASS-concentrated region of plasma membrane were measured in the presence of dasatinib. The graph shows percentages of cells showing that more than 50% of Ral-GDS was localized in each region (*n* = 30). (**C**) (Left upper) Displacement of scramble or C3G knockdown cells were measured on ICAM-1 with or without CXCL12 (*n* = 30). (lower) Representative tracks of scramble or C3G KD cells are shown. Each line represents a single-cell track. (Right upper) Co-localization of PASS-GFP and Ral-GDS-RBD-mCherry in C3G KD cells after CXCL12 stimulation is shown. (lower) The ratios of Ral-GDS localized in the cytoplasm, plasma membrane and PASS-concentrated region of plasma membrane were measured. The graph shows percentages of cells showing that more than 50% of Ral-GDS was localized in each region (*n* = 30). (**D**) (Left) Immunoblots with anti-C3G of cell lysates from scramble or C3G KD BAF cells. Actin is a loading control. (Right) Scramble and C3G KD cells were stimulated with CXCL12 at the indicated times, and subjected to the pull-down assay. Bound Rap1 (Rap1-GTP) and total Rap1 were detected with anti-Rap1. Each bar graph represents the means ±SEM. Scale bar, 5 μm.

**Additional file 4: Figure S4.** Effects of phosphorylation/de-phosphorylation of RA-GEF. (**A**) Prevention of T-cell migration by the inhibition of de-phosphorylation. (Top) The displacement and velocity of WT T cells on ICAM-1 were measured in the presence or absence of CCL21, with or without OA (*n* = 30). *^1^*P* < 0.001, *^2^*P* < 0.005 versus CCL21-stimulated cells without OA. (Bottom) The representative tracks of WT T cells with CCL21 are shown. Each line represents a single-cell track. (**B**) Reduced CXCL12-induced Rap1 activation by overexpression of WNK1. Control or WNK1-expressing BAF cells were stimulated with CXCL12, and subjected to a pull-down assay. Bound Rap1 (Rap1-GTP) and total Rap1 were detected with anti-Rap1. (**C**) Control or WNK1-expressing BAF cells stimulated with or without CXCL12 for 60 s in the presence or absence of OA, was analyzed by Phos-tag (upper) or conventional (lower) SDS-PAGE followed by immunoblotting with anti-RA-GEF-2. (**D**) Increased CXCL12-induced Rap1 activation by the knockdown of WNK1. (Top and middle) Control or WNK1 KD BAF cells were stimulated with CXCL12 and subjected to a pull-down assay. Bound Rap1 (Rap1-GTP) and total Rap1 were detected with anti-Rap1. Representative two blots from three independent experiments are shown (Exp. I and II). (Bottom) Quantification of Rap1-GTP is presented as fold increase of Rap1-GTP in control or WNK1 KD cells at times after CXCL12 stimulation relative to unstimulated control cells (adjusted to 1). Each point represents the means ±SEM from three independent experiments. *P < 0.001 versus control cells. (**E**) Distribution of flag-RA-GEF-1 (red) and CD44 (green) in control and WNK1 KD cells that were untreated or treated with CXCL12 for 10 min is shown. Scale bar, 5 μm. (Bottom) The graph shows the percentages of cells with the polarized membrane localization of flag-RA-GEF-1 in opposite site of CD44. (*n* = 30). Each bar graph represents the means ±SEM.

**Additional file 5: Table S1.** Individual data in graphs of Fig. 1D, Fig. 2B, C, Fig. 3A, B, Fig. 4A, B, C, D, Fig. 7, Fig. 8 and Fig. S4. The graphs in Fig. 1D**,** Fig. 2B, C, Fig. 3A, B, Fig. 4A, B, C, D show the averages +SEM of three or five independent experiments. The graphs in Figs. 7 and 8 show the averages +SEM of the percentages of each ①, ② and ③ band of RA-GEF-1 or RA-GEF-2 from three independent experiments. The graphs in Fig. S4 show the averages +SEM of the fold increase of the abundance of Rap-1-GTP with CXCL12 stimulation from three independent experiments.

## Data Availability

All relevant data are within the paper and its supporting Additional files.

## Abbreviations

RA-GEF: Ras/Rap association-guanine nucleotide exchange factor
RA-GEF-1: also termed PDZ-GEF1, Rapgef2)
RA-GEF-2: also termed PDZ-GEF2, Rapgef6

## References

[1] Springer TA (1994). Traffic signals for lymphocyte recirculation and leukocyte emigration: the multistep paradigm. Cell.

[2] Katagiri K, Hattori M, Minato N, Kinashi T (2002). Rap1 functions as a key regulator of T-cell and antigen-presenting cell interactions and modulates T-cell responses. Mol Cell Biol.

[3] Ebisuno Y, Katagiri K, Katakai T, Ueda Y, Nemoto T, Inada H, Nabekura J, Okada T, Kannagi R, Tanaka T, Miyasaka M, Hogg N, Kinashi T (2009). Rap1 controls lymphocyte adhesion cascade and interstitial migration within lymph nodes in RAPL-dependent and -independent manners. Blood.

[4] Katagiri K, Maeda A, Shimonaka M, Kinashi T (2003). RAPL, a Rap1-binding molecule that mediates Rap1-induced adhesion through spatial regulation of LFA-1. Nat Immunol.

[5] Katagiri K, Katakai T, Ebisuno Y, Ueda Y, Okada T, Kinashi T (2009). Mst1 controls lymphocyte trafficking and interstitial motility within lymph nodes. EMBO J.

[6] Ishihara S, Nishikimi A, Umemoto E, Miyasaka M, Saegusa M, Katagiri K (2015). Dual functions of Rap1 are crucial for T-cell homeostasis and prevention of spontaneous colitis. Nat Commun.

[7] Baeyens A, Fang V, Chen C, Schwab SR (2015). Exit strategies: S1P signaling and T cell migration. Trends Immunol.

[8] Cyster JG, Schwab SR (2012). Sphingosine-1-phosphate and lymphocyte egress from lymphoid organs. Annu Rev Immunol.

[9] Garris CS, Blaho VA, Hla T, Han MH, Sphingosine-1-phosphate receptor 1 signalling in T cells: trafficking and beyond, Immunology, 2014;142:347–353. 10.1111/imm.12272 PMC4080950 24597601

[10] Katagiri K, Ohnishi N, Kabashima K, Iyoda T, Takeda N, Shinkai Y, Inaba K, Kinashi T (2004). Crucial functions of the Rap1 effector molecule RAPL in lymphocyte and dendritic cell trafficking. Nat Immunol.

[11] Mou F, Praskova M, Xia F, Van Buren D, Hock H, Avruch J, Zhou D (2012). The Mst1 and Mst2 kinases control activation of rho family GTPases and thymic egress of mature thymocytes. J Exp Med.

[12] Gloerich M, Bos JL (2011). Regulating Rap small G-proteins in time and space. Trends Cell Biol.

[13] Gu JJ, Lavau CP, Pugacheva E, Soderblom EJ, Moseley MA, Pendergast AM (2012). Abl family kinases modulate T cell-mediated inflammation and chemokine-induced migration through the adaptor HEF1 and the GTPase Rap1. Sci Signal.

[14] Huang Y, Clarke F, Karimi M, Roy NH, Williamson EK, Okumura M, Mochizuki K, Chen EJ, Park TJ, Debes GF, Zhang Y, Curran T, Kambayashi T, Burkhardt JK (2015). CRK proteins selectively regulate T cell migration into inflamed tissues. J Clin Invest.

[15] Malherbe LP, Wang D (2012). Tyrosine kinases EnAbling adaptor molecules for chemokine-induced Rap1 activation in T cells. Sci Signal.

[16] Montresor A, Bolomini-Vittori M, Toffali L, Rossi B, Constantin G, Laudanna C (2013). JAK tyrosine kinases promote hierarchical activation of Rho and Rap modules of integrin activation. J Cell Biol.

[17] Bilasy SE, Satoh T, Ueda S, Wei P, Kanemura H, Aiba A, Terashima T, Kataoka T (2009). Dorsal telencephalon-specific RA-GEF-1 knockout mice develop heterotopic cortical mass and commissural fiber defect. Eur J Neurosci.

[18] Maeta K, Edamatsu H, Nishihara K, Ikutomo J, Bilasy SE, Kataoka T. Crucial role of Rapgef2 and Rapgef6, a family of guanine nucleotide exchange factors for Rap1 small GTPase, in formation of apical surface adherens junctions and neural progenitor development in the mouse cerebral cortex. eNeuro, 2016;3:ENEURO0142–16. 10.1523/ENEURO.0142-16.2016 PMC4917737 27390776

[19] Yoshikawa Y, Satoh T, Tamura T, Wei P, Bilasy SE, Edamatsu H, Aiba A, Katagiri K, Kinashi T, Nakao K, Kataoka T (2007). The M-Ras-RA-GEF-2-Rap1 pathway mediates tumor necrosis factor-alpha dependent regulation of integrin activation in splenocytes. Mol Biol Cell.

[20] Consonni SV, Brouwer PM, van Slobbe ES, Bos JL (2014). The PDZ domain of the guanine nucleotide exchange factor PDZGEF directs binding to phosphatidic acid during brush border formation. PLoS One.

[21] Momoi Y, Nishikimi A, Du G, Kataoka T, Katagiri K. Phosphatidic acid regulates subcellular distribution of RA-GEFs critical for chemokine-dependent migration. Biochem Biophys Res Commun. 2020;524:325–331. 10.1016/j.bbrc.2020.01.080 31996307

[22] Zhang F, Wang Z, Lu M, Yonekubo Y, Liang X, Zhang Y, Wu P, Zhou Y, Grinstein S, Hancock JF, Du G (2014). Temporal production of the signaling lipid phosphatidic acid by phospholipase D2 determines the output of extracellular signal-regulated kinase signaling in cancer cells. Mol Cell Biol.

[23] Du G, Huang P, Liang BT, Frohman MA (2004). Phospholipase D2 localizes to the plasma membrane and regulates angiotensin II receptor endocytosis. Mol Biol Cell.

[24] Xu X, Zhang S, Li P, Lu J, Xuan Q, Ge Q (2013). Maturation and emigration of single-positive thymocytes. Clin Dev Immunol.

[25] Beck TC, Gomes AC, Cyster JG, Pereira JP (2014). CXCR4 and a cell-extrinsic mechanism control immature B lymphocyte egress from bone marrow. J Exp Med.

[26] Tsuneto M, Tokoyoda K, Kajikhina E, Hauser AE, Hara T, Tani-Ichi S, Ikuta K, Melchers F (2013). B-cell progenitors and precursors change their microenvironment in fetal liver during early development. Stem Cells.

[27] Nishikimi A, Ishihara S, Ozawa M, Etoh K, Fukuda M, Kinashi T, Katagiri K (2014). Rab13 acts downstream of the kinase Mst1 to deliver the integrin LFA-1 to the cell surface for lymphocyte trafficking. Sci Signal.

[28] Jeon TJ, Lee DJ, Lee S, Weeks G, Firtel RA (2007). Regulation of Rap1 activity by RapGAP1 controls cell adhesion at the front of chemotaxing cells. J Cell Biol.

[29] Nolz JC, Nacusi LP, Segovis CM, Medeiros RB, Mitchell JS, Shimizu Y, Billadeau DD (2008). The WAVE2 complex regulates T cell receptor signaling to integrins via Abl- and CrkL-C3G-mediated activation of Rap1. J Cell Biol.

[30] Sakakibara A, Ohba Y, Kurokawa K, Matsuda M, Hattori S (2002). Novel function of Chat in controlling cell adhesion via Cas-Crk-C3G-pathway-mediated Rap1 activation. J Cell Sci.

[31] Letschka T, Kollmann V, Pfeifhofer-Obermair C, Lutz-Nicoladoni C, Obermair GJ, Fresser F, Leitges M, Hermann-Kleiter N, Kaminski S, Baier G (2008). PKC-theta selectively controls the adhesion-stimulating molecule Rap1. Blood.

[32] Utreras E, Henriquez D, Contreras-Vallejos E, Olmos C, Di Genova A, Maass A, Kulkarni AB, Gonzalez-Billault C (2013). Cdk5 regulates Rap1 activity. Neurochem Int.

[33] Kochl R, Thelen F, Vanes L, Brazao TF, Fountain K, Xie J, Huang CL, Lyck R, Stein JV, Tybulewicz VL. WNK1 kinase balances T cell adhesion versus migration in vivo. Nat Immunol. 2016;17:1075–1083. 10.1038/ni.3495 PMC4994873 27400149

[34] Crittenden JR, Bergmeier W, Zhang Y, Piffath CL, Liang Y, Wagner DD, Housman DE, Graybiel AM (2004). CalDAG-GEFI integrates signaling for platelet aggregation and thrombus formation. Nat Med.

[35] Katagiri K, Imamura M, Kinashi T (2006). Spatiotemporal regulation of the kinase Mst1 by binding protein RAPL is critical for lymphocyte polarity and adhesion. Nat Immunol.

[36] Shimonaka M, Katagiri K, Nakayama T, Fujita N, Tsuruo T, Yoshie O, Kinashi T (2003). Rap1 translates chemokine signals to integrin activation, cell polarization, and motility across vascular endothelium under flow. J Cell Biol.

[37] Kochl R, Thelen F, Vanes L, Brazao TF, Fountain K, Xie J, Huang CL, Lyck R, Stein JV, Tybulewicz VL (2017). Corrigendum: WNK1 kinase balances T cell adhesion versus migration in vivo. Nat Immunol.

[38] Katagiri K, Ueda Y, Tomiyama T, Yasuda K, Toda Y, Ikehara S, Nakayama KI, Kinashi T. Deficiency of Rap1-binding protein RAPL causes lymphoproliferative disorders through mislocalization of p27kip1. Immunity 2011;34:24–38. 10.1016/j.immuni.2010.12.010 21194982

[39] Ueda Y, Katagiri K, Tomiyama T, Yasuda K, Habiro K, Katakai T, Ikehara S, Matsumoto M, Kinashi T (2012). Mst1 regulates integrin-dependent thymocyte trafficking and antigen recognition in the thymus. Nat Commun.

[40] Katagiri K, Hattori M, Minato N, Irie S, Takatsu K, Kinashi T (2000). Rap1 is a potent activation signal for leukocyte function-associated antigen 1 distinct from protein kinase C and phosphatidylinositol-3-OH kinase. Mol Cell Biol.

[41] Katagiri K, Shimonaka M, Kinashi T (2004). Rap1-mediated lymphocyte function-associated antigen-1 activation by the T cell antigen receptor is dependent on phospholipase C-gamma1. J Biol Chem.

[42] Fukujin F, Nakajima A, Shimada N, Sawai S. Self-organization of chemoattractant waves in Dictyostelium depends on F-actin and cell-substrate adhesion. J R Soc Interface 2016;13:20160233. 10.1098/rsif.2016.0233 PMC4938087 27358278

[43] Nakajima A, Ishida M, Fujimori T, Wakamoto Y, Sawai S (2016). The microfluidic lighthouse: an omnidirectional gradient generator. Lab Chip.

