# Supplementary figures and images for: Phosphatidic acid-dependent localization and basal de-phosphorylation of RA-GEFs regulate lymphocyte trafficking
Source: BMC Biol. 2020 Jun 29;18:75. doi: 10.1186/s12915-020-00809-0 (PMC7325102; doi:10.1186/s12915-020-00809-0)

Supplementary Fig.1

A

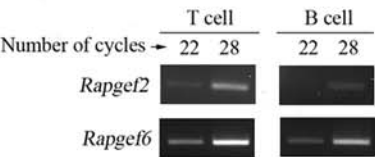

B

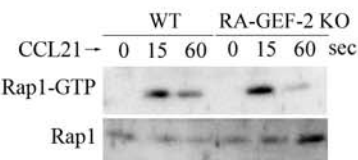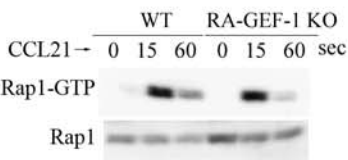

Supplement: Supplementary file 1 — Additional file 1: Figure S1.Rapgef2 and 6 in T cells. (A) Expression analysis of Rapgef2 and 6 in T and B cells by RT-PCR. RNA isolated from purified T cells and B cells was reverse transcribed and used as template for PCR amplification. PCR reactions were performed using each specific primer under optimal conditions. (B) Effect of Rapgef2 or 6 deficiency on CCL21-Rap1 activation in T cells. GTP-bound Rap1 was analyzed by a pull-down assay using GST-RalGDS-RBD. RA-GEF-1 (right) or RA-GEF-2 (left)-deficient mouse T cells stimulated with 100 nM of CCL21 at the indicated times, lysed and subjected to a pull-down assay. Bound Rap1 and total Rap1 were detected by immunoblotting with an anti-Rap1 antibody. [file 12915_2020_809_MOESM1_ESM.pdf]

## Supplementary Fig.2

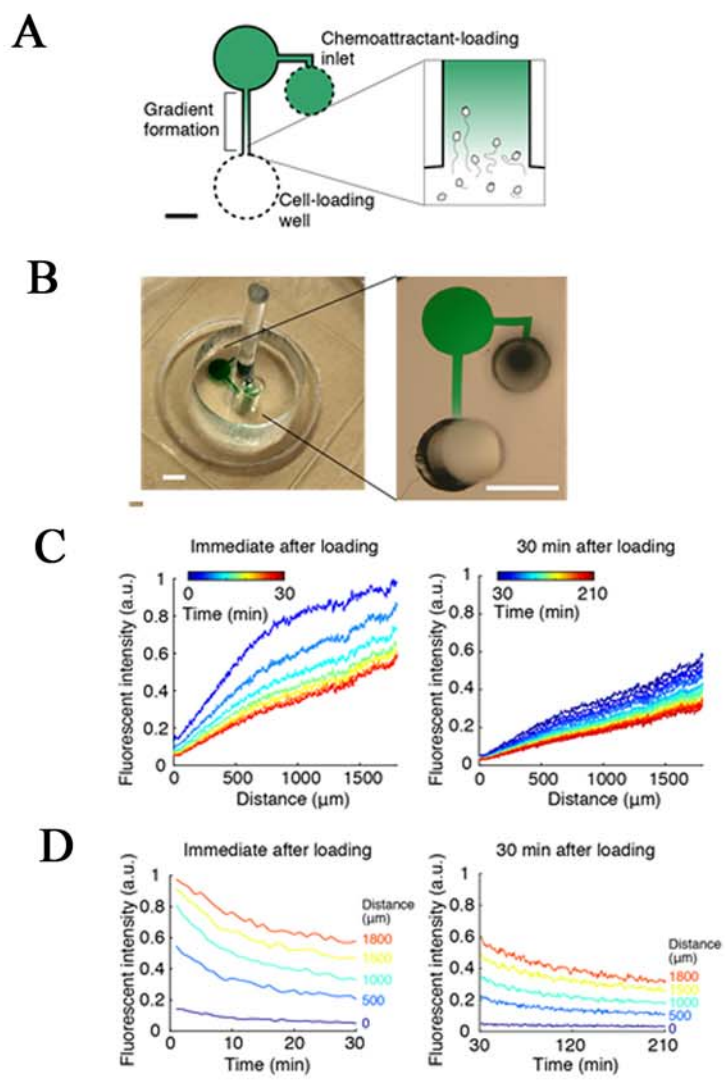

Supplement: Supplementary file 2 — Additional file 2: Figure S2. Gradient formation in the chemotaxis chamber. (A) A chamber overview. The chamber consists of a block of molded PDMS bonded to a glass-bottom dish that together form a 2 mm long linear channel. The height and the width of the channel are 50 μm and 250 μm, respectively. One side of the channel is connected to a cell-loading well, and the other side is connected to a chemoattractant circular reservoir of a diameter 2 mm. The reservoir is connected to an inlet for chemoattractant loading though a 200 μm-wide channel. The concentration gradient is formed by passive diffusion (green) by closing the inlet with a plug. Scale bar, 1 μm. (B) Snapshots of the concentration gradient in the chemotaxis chamber where a green food coloring was loaded for demonstration. Scale bar, 2 mm. (C) Evaluation of the gradient profile; PBS containing 10 μM fluorescein was loaded for visualization. The time-course of the fluorescent intensity profiles during the initial transient (left panel; immediately after loading) and after the initial transient (right panel; 30 min after loading). The mean fluorescence intensities of a 250 μm wide area as a function of the distance from the border between the channel and the cell-loading well; data was plotted every 5 min (left panel) and 10 min (right panel), respectively. (D) The time-course of the fluorescent intensity. The fluorescent intensity at 0 μm (dark blue), 500 μm (blue), 1000 μm (cyan), 1500 μm (yellow), and 1800 μm (red) from the cell-loading well during the initial transient (left panel; immediately after loading) and chemotaxis assay (right panel; 30 min after loading). [file 12915_2020_809_MOESM2_ESM.pdf]

Supplementary Fig.3

A

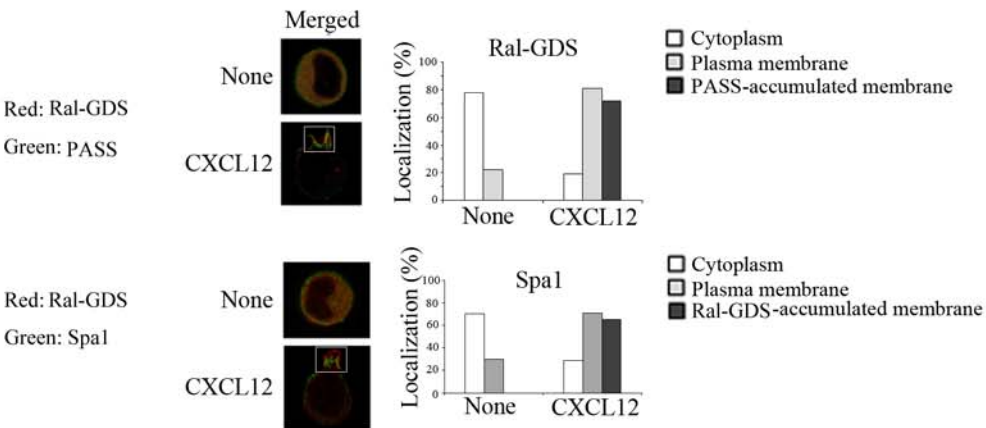

B

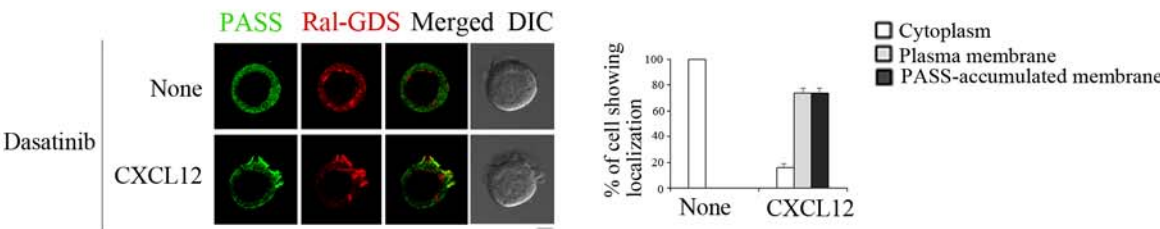

C

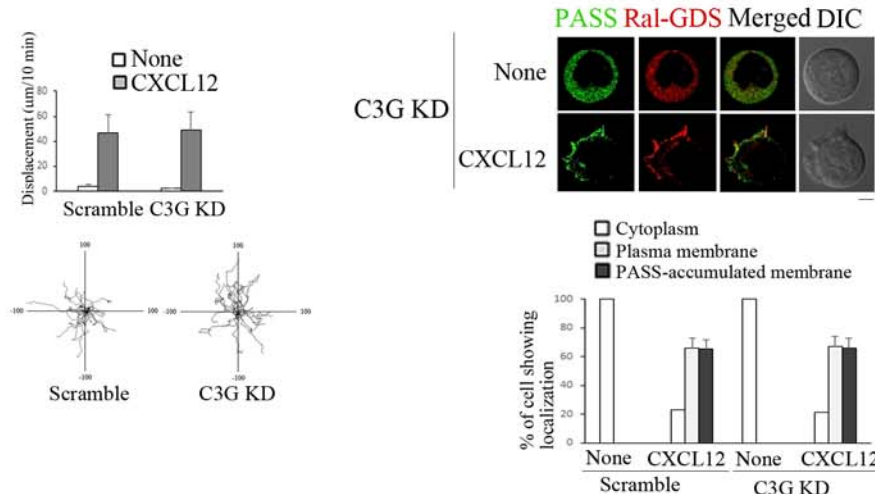

D

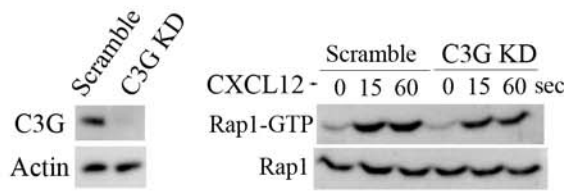

Supplement: Supplementary file 3 — Additional file 3: Figure S3. Localization of PA-dependent Rap1-GTP in the front membrane during cell migration. (A) (Top left) We measured the ratios of Ral-GDS (red) in the cytoplasm, plasma membrane and PASS (green)-concentrated region of plasma membrane (white rectangular) in unstimulated and CXCL12-stimulated cells. (right) The graph shows ratios of Ral-GDS localized in each region (cytoplasm, plasma membrane or PASS-concentrated membrane). (Bottom left) We measured the ratios of Spa1 (green) in the cytoplasm, plasma membrane and Ral-GDS (red)-concentrated region of plasma membrane (white rectangular) in unstimulated and CXCL12-stimulated cells. (right) The graph shows ratios of Spa1 localized in each region. (B) (Left) Co-localization of PASS-GFP and Ral-GDS-RBD-mCherry in BAF cells after CXCL12 stimulation in the presence of dasatinib is shown. (Right) The ratios of Ral-GDS localized in the cytoplasm, plasma membrane and PASS-concentrated region of plasma membrane were measured in the presence of dasatinib. The graph shows percentages of cells showing that more than 50% of Ral-GDS was localized in each region (n = 30). (C) (Left upper) Displacement of scramble or C3G knockdown cells were measured on ICAM-1 with or without CXCL12 (n = 30). (lower) Representative tracks of scramble or C3G KD cells are shown. Each line represents a single-cell track. (Right upper) Co-localization of PASS-GFP and Ral-GDS-RBD-mCherry in C3G KD cells after CXCL12 stimulation is shown. (lower) The ratios of Ral-GDS localized in the cytoplasm, plasma membrane and PASS-concentrated region of plasma membrane were measured. The graph shows percentages of cells showing that more than 50% of Ral-GDS was localized in each region (n = 30). (D) (Left) Immunoblots with anti-C3G of cell lysates from scramble or C3G KD BAF cells. Actin is a loading control. (Right) Scramble and C3G KD cells were stimulated with CXCL12 at the indicated times, and subjected to the pull-down assay. Bound Rap1 (Rap [file 12915_2020_809_MOESM3_ESM.pdf]

# Supplementary Fig.4

A

T cells

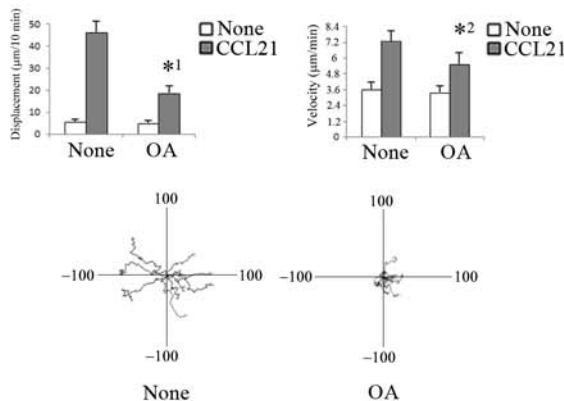

B

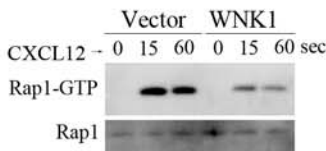

C

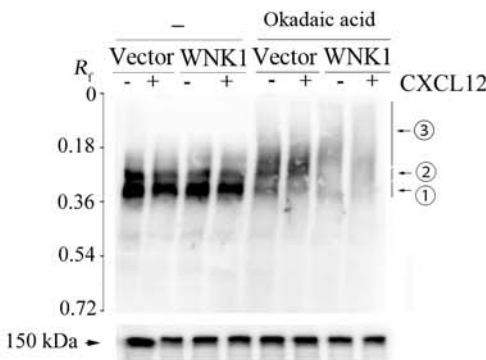

D

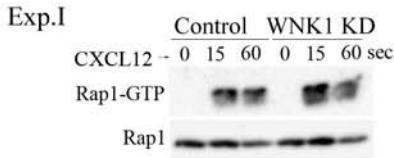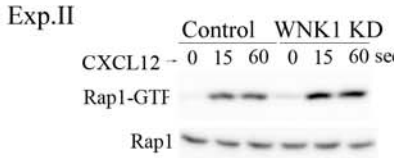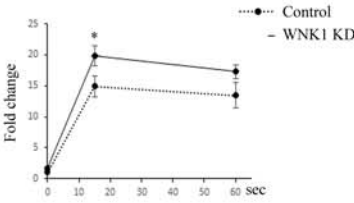

E

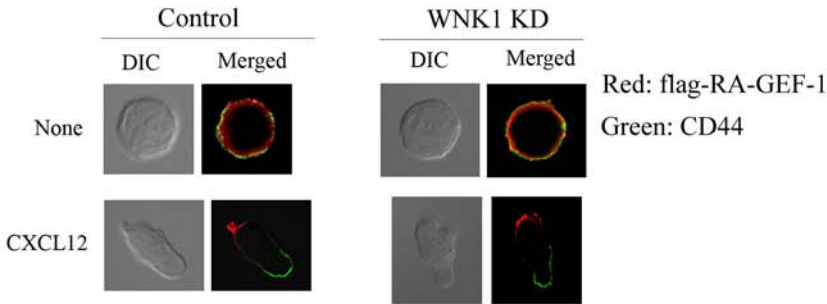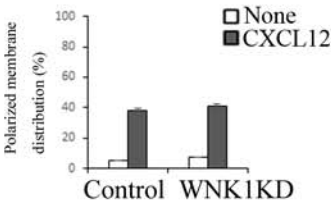

Supplement: Supplementary file 4 — Additional file 4: Figure S4. Effects of phosphorylation/de-phosphorylation of RA-GEF. (A) Prevention of T-cell migration by the inhibition of de-phosphorylation. (Top) The displacement and velocity of WT T cells on ICAM-1 were measured in the presence or absence of CCL21, with or without OA (n = 30). *1P < 0.001, *2P < 0.005 versus CCL21-stimulated cells without OA. (Bottom) The representative tracks of WT T cells with CCL21 are shown. Each line represents a single-cell track. (B) Reduced CXCL12-induced Rap1 activation by overexpression of WNK1. Control or WNK1-expressing BAF cells were stimulated with CXCL12, and subjected to a pull-down assay. Bound Rap1 (Rap1-GTP) and total Rap1 were detected with anti-Rap1. (C) Control or WNK1-expressing BAF cells stimulated with or without CXCL12 for 60 s in the presence or absence of OA, was analyzed by Phos-tag (upper) or conventional (lower) SDS-PAGE followed by immunoblotting with anti-RA-GEF-2. (D) Increased CXCL12-induced Rap1 activation by the knockdown of WNK1. (Top and middle) Control or WNK1 KD BAF cells were stimulated with CXCL12 and subjected to a pull-down assay. Bound Rap1 (Rap1-GTP) and total Rap1 were detected with anti-Rap1. Representative two blots from three independent experiments are shown (Exp. I and II). (Bottom) Quantification of Rap1-GTP is presented as fold increase of Rap1-GTP in control or WNK1 KD cells at times after CXCL12 stimulation relative to unstimulated control cells (adjusted to 1). Each point represents the means ±SEM from three independent experiments. *P < 0.001 versus control cells. (E) Distribution of flag-RA-GEF-1 (red) and CD44 (green) in control and WNK1 KD cells that were untreated or treated with CXCL12 for 10 min is shown. Scale bar, 5 μm. (Bottom) The graph shows the percentages of cells with the polarized membrane localization of flag-RA-GEF-1 in opposite site of CD44. (n = 30). Each bar graph represents the means ±SEM. [file 12915_2020_809_MOESM4_ESM.pdf]
